# Inverse ceria-nickel catalyst for enhanced C–O bond hydrogenolysis of biomass and polyether

**DOI:** 10.1038/s41467-024-52704-9

**Published:** 2024-09-30

**Authors:** Zelun Zhao, Guang Gao, Yongjie Xi, Jia Wang, Peng Sun, Qi Liu, Chengyang Li, Zhiwei Huang, Fuwei Li

**Affiliations:** 1 State Key Laboratory of Low Carbon Catalysis and Carbon Dioxide Utilization, State Key Laboratory for Oxo Synthesis and Selective Oxidation, Lanzhou Institute of Chemical Physics, Chinese Academy of Sciences, Lanzhou, 730000 China; 2 School of Chemical Engineering, University of Chinese Academy of Sciences, Beijing, 100049 China

**Keywords:** Heterogeneous catalysis, Sustainability, Polymer chemistry

## Abstract

Regulating interfacial electronic structure of oxide-metal composite catalyst for the selective transformation of biomass or plastic waste into high-value chemicals through specific C–O bond scission is still challenging due to the presence of multiple reducible bonds and low catalytic activity. Herein, we find that the inverse catalyst of 4CeO_x_/Ni can efficiently transform various lignocellulose derivatives and polyether into the corresponding value-added hydroxyl-containing chemicals with activity enhancement (up to 36.5-fold increase in rate) compared to the conventional metal/oxide supported catalyst. In situ experiments and theoretical calculations reveal the electron-rich interfacial Ce and Ni species are responsible for the selective adsorption of C–O bond and efficient generation of H^δ−^ species, respectively, which synergistic facilitate cleavage of C–O bond and subsequent hydrogenation. This work advances the fundamental understanding of interfacial electronic interaction over inverse catalyst and provides a promising catalyst design strategy for efficient transformation of C–O bond.

## Abstract (web summary)

Selective C-O bond scission is a challenging process for the transformation of biomass or plastic waste. This study reports an inverse ceria nickel catalyst showcasing enhanced hydrogenolysis activity and selectivity.

## Introduction

The strong interface electronic interaction in a metal-oxide heterogeneous catalyst is essential in deciding its catalytic properties by tuning the adsorption and activation of adsorbed reactants and/or intermediates over the catalyst surface, where the distribution of interface electrons can be rearranged through charge transfer driven by the difference in the Fermi level of metal and oxide^1,2^. Interestingly, changing catalyst configuration from conventional supported metal catalyst over oxide support to the inverse oxide on metal has been employed as a promising tool for modulating the surface electronic state and found wide applications with enhanced catalytic activity and selectivity, particularly in the catalytic conversion of small molecules, such as CO_2_, CO, and O_2_^3–7^. Notably, the inverse ZrO_2_/Cu catalyst demonstrates 3.3 times higher activity compared to traditional Cu/ZrO_2_ catalyst in the hydrogenation of CO_2_ to methanol, since the strong electronic interaction induced interface Zr active sites on inverse catalyst promote the adsorption of CO_2_ and the subsequent formation of formate intermediates, which lead to the enhanced activity of CO_2_ hydrogenation^3^. Moreover, the strong oxide-metal interaction facilitates the charge transfer from the metal Pd to oxide RuO_x_ in the inverse RuO_x_/Pd catalyst and, therefore, changes the adsorption configuration of O_2_ molecule, resulting in a 24-fold enhancement in the activity of oxygen reduction reaction compared to the Pd/C catalyst^4^. Nevertheless, the exploring application of inverse catalyst in the precise transformation of complex molecules and even polymer has been much less conducted, due to the challenges on selectively tuning the adsorption and activation of different functional groups^8–11^.

Biomass-derived molecules and oxygen-containing polymer wastes have varied C–O bonds, such as etheric/furanic C–O bond in (hemi)cellulose derivates^12^, phenolic C–O bond in lignin components^8^, C(alkyl)–O bond in polyether^13^, therefore, upgrading of biomass and recycling of plastic wastes through selective hydrogenolysis of specific C–O bond into value-added oxygenates with high atom-efficiency is a promising strategy towards carbon neutrality^14,15^. For example, ring-opening hydrogenation of tetrahydrofurfuryl alcohol (THFA) into 1,5–pentanediol (1,5–PDO) via selective cleavage of etheric C–O bond is a sustainable process for the synthesis of C5 diols compared to their production from petroleum feedstock at the industrial scale^16,17^. Although the development of noble metal (Ir, Pd, Ru, and Rh) catalysts efficiently improved the catalytic performance of the reaction by introducing MO_x_ (M = Re, Mo, W, and V) in the last decades^18–23^, it was found that the promotor MO_x_ boosted the further hydrodeoxygenation of 1,5–PDO to the byproduct of 1–pentanol, resulting in that the reaction gave ideal selectivity to 1,5–PDO under low conversion and/or concentration of THFA^24–26^. Moreover, the high cost of catalysts and stability deterioration due to the leaching of MO_x_ limit their application^16,27^. Unfortunately, these limitations become even more serious over recently developed Ni-based heterogeneous catalysts (Supplementary Table 1)^28–32^. Therefore, it is highly desirable to develop an efficient and long-lifetime non-precious metal catalyst for the hydrogenolysis of THFA, and hopefully for the other oxygenates containing etheric C–O bond as well.

Enlightened by effective tuning of strong electronic interaction on inverse oxide/metal catalyst and the resultant enhancement in catalytic performance, in this work, the ceria-nickel catalysts with inverse oxide/metal configuration were prepared by co-precipitation method. The inverse 4CeO_x_/Ni afforded a 98% 1,5–PDO selectivity under 95% conversion in the ring-opening hydrogenation of THFA, and demonstrated a promising catalytic activity of 36.5-fold in the reactive rate compared with conventional 96CeO_x_/Ni catalyst. Moreover, this inverse catalyst exhibited exceptional stability during a 200-hour continuous reaction, even in the absence of any solvent beyond the general requirement of a low concentration of reactant in the reported literature. The structure analysis of catalysts revealed the electronic state of the interfacial Ce and Ni species could be modulated by building the inverse oxide/metal configuration due to the strong electronic interaction. Further, in situ infrared Fourier transform (FT-IR) experiments and density functional theory (DFT) calculation results disclosed both the selective adsorption of etheric oxygen in THFA at the interface Ce sites adjacent to oxygen vacancy and the enhanced formation of H^δ−^ species at the interface Ni sites contributed to the superior catalytic performance of C–O bond hydrogenolysis. Interestingly, the inverse 4CeO_x_/Ni catalyst also exhibited enhancement in different etheric C–O bonds hydrogenolysis of the other representative biomass derivates and polyether to the desired alcohols. This work provides a promising example of boosting the non-precious metal-catalyzed utilization of biomass and oxygen-containing polymer waste to valuable hydroxyl compounds by tuning the interfacial electronic structure over inverse catalysts beyond conventional supported metal-oxide catalysts.

## Results

### Development of efficient and stable inverse Ce-Ni catalyst for THFA hydrogenation to 1,5–PDO

A coprecipitation method was employed to prepare a series of supported Ce-Ni catalysts^3^, which were denoted as nCeO_x_/Ni (*n* = 1, 4, 85, and 96) according to the Ce loadings determined by inductively coupled plasma mass spectrometry (ICP-MS) as shown in Supplementary Table 2. Using X-ray powder diffraction (XRD), the remarkable Ni crystal phase (PDF No: 65–2865) was observed in the 4CeO_x_/Ni and 1CeO_x_/Ni catalysts (Supplementary Fig. 1), and the negligible CeO_2_ diffraction peaks could be ascribed to both the low content of Ce and the high dispersion of CeO_x_ cluster^33^. In contrast, the strong diffraction peaks observed in the 96CeO_x_/Ni and 85CeO_x_/Ni catalysts indicated the presence of the characteristic fluorite CeO_2_ phase (PDF No: 65–5923). Their great contrast in compositions suggested the different structural configurations of Ni and CeO_2_ in the nCeO_x_/Ni catalysts, whose geometrical structure was studied by high-resolution transmission electron microscopy, high-angle annular dark field scanning transmission electron microscopy, and energy-dispersive X-ray spectroscopy. As shown in Supplementary Fig. 2a, a representative inverse oxide/metal configuration of well-dispersed nano CeO_x_ clusters (size of ~3 nm) supported on the Ni surface was observed over the 4CeO_x_/Ni catalyst. In contrast, the 96CeO_x_/Ni catalyst has a typical metal/oxide configuration, where the Ni particles (size of ~6 nm) were supported on the bulk CeO_2_ (Supplementary Fig. 2b). Their geometrical structures were further studied by using element analysis through in situ X-ray photoelectron spectroscopy (XPS) combined with Ar^+^ ion sputtering treatment (Supplementary Fig. 3). The intensity of Ni peak at 852.3 eV for inverse 4CeO_x_/Ni catalyst increased from 2.0 × 10^5^ to 4.2 × 10^5^ counts after 100 s of sputtering. Simultaneously, the intensity of the Ce peak at 885.1 eV diminished from 1.6 × 10^4^ to 0.7 × 10^4^ counts. In contrast, after sputtering, the enhancement of Ce signals and the weakness of Ni signals were observed over conventional 96CeO_x_/Ni catalyst as the erosion depth increased. These results demonstrated that Ce species were scattered on bulk Ni surface of inverse 4CeO_x_/Ni catalyst, while Ni species were distributed on the CeO_2_ surface of conventional 96CeO_x_/Ni catalyst.

**Fig. 1:**
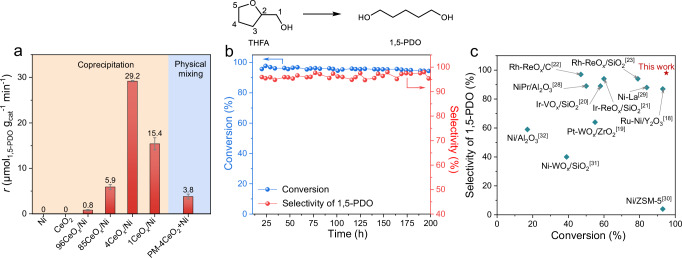
Transformation of THFA into 1,5–PDO via etheric C–O bond hydrogenolysis. **a** Catalytic performance over Ce-Ni catalysts. Reaction conditions: calcined catalyst: 5.4 g; THFA (solvent-free): 0.02 mL min^−1^; 160 °C; 4.0 MPa H_2_; gas-flow rate: 50 mL min^−1^. Error bars refer to the standard deviation based on three measurements. **b** Catalyst stability experiment in a fixed-bed reactor over a 4CeO_x_/Ni catalyst, calcined catalyst: 13 g, Red: selectivity of 1,5–PDO, blue: conversion of THFA. Before the reaction, the calcined catalyst was pre-reduced in an H_2_/N_2_ flow. **c** THFA hydrogenolysis over the representative supported catalysts^18–23,28–32^. THFA: tetrahydrofurfuryl alcohol, 1,5–PDO: 1,5–pentanediol.

To develop an efficient non-precious metal catalyst for the sustainable upgrading of oxygen-containing biomass and plastic wastes, the ring-opening hydrogenation of THFA, derived from hemicellulose-based furfural, into 1,5–PDO was initially selected as a probing reaction. The inverse 4CeO_x_/Ni catalyst presented the highest production rate of 29.2 μmol_1,5–PDO_ g_cat_^−1^ min^−1^ under controlled THFA conversion of 63% (1,5–PDO selectivity of 98%) in a continuous flow fixed-bed reactor, which was 36.5 times than that of 0.8 μmol_1,5–PDO_ g_cat_^−1^ min^−1^ obtained over conventional 96CeO_x_/Ni catalyst (Fig. 1a, Supplementary Table 3). The physical mixed catalyst (PM-4CeO_2_ + Ni) with the same molar ratio of Ce and Ni as 4CeO_x_/Ni catalyst produced a much lower 1,5–PDO production rate of 3.8 μmol_1,5–PDO_ g_cat_^−1^ min^−1^. In addition, trace or no 1,5–PDO was obtained over bulk Ni or CeO_2_ alone. These results indicated that the interfacial Ce-Ni sites over inverse 4CeO_x_/Ni catalysts is responsible for the hydrogenolysis of C–O bond, where the synergy between Ce and Ni species is highly essential in the ring-opening hydrogenation of THFA^34^. To provide more information about the relationship between C–O bond hydrogenolysis activity and interface Ce-Ni sites, the reaction rate was normalized by their interfacial perimeter length, surface area, Ce mass, and Ni mass (Supplementary Fig. 4), which generally demonstrated the representative inverse 4CeO_x_/Ni catalyst exhibited superior catalytic activity compared to the representative conventional 96CeO_x_/Ni catalyst^33,35^. The carbon monoxide temperature-programmed desorption combined with mass spectrometry (CO-TPD-MS) results (Supplementary Fig. 5) of nCeO_x_/Ni catalysts indicated a remarkable difference in the surface metallic sites between inverse and conventional catalyst because of the negligible CO desorption for the 4CeO_x_/Ni catalysts, compared to the strong desorption peaks at 250 °C and 600 °C for the 96CeO_x_/Ni catalyst^36^. Moreover, the oxidation of CO into CO_2_ by CeO_x_ species occurred at a lower temperature of 200 °C over the 4CeO_x_/Ni catalysts, as compared to the temperature of 550 °C required over the 96CeO_x_/Ni catalyst, which could be ascribed to the weakened Ce–O bond in inverse catalyst^37^. Based on the above results, it could be preliminarily deduced that the difference in the geometrical and electronic structure of inverse and conventional interfacial Ce-Ni sites contributed to the superior catalytic activity of the inverse catalyst compared to its conventional counterpart in the THFA hydrogenolysis.

Due to the unideal hydrodeoxygenation of 1,5–PDO to 1–pentanol over the catalysts with promoters such as ReO_x_, MoO_x_, WO_x_, and VO_x_ (Supplementary Table 4), the controlled low conversion and using low concentration of substrate were necessary to achieve high selectivity of 1,5–PDO^24,25^. To our delight, under the high conversion of THFA above 95% and without using any solvent, 98% selectivity of 1,5–PDO was obtained over inverse 4CeO_x_/Ni catalyst (Fig. 1b). Those results are much better than the reported data obtained over even noble catalysts up to now (Fig. 1c and Supplementary Table 1)^18–23,28–32^. Notably, the inverse 4CeO_x_/Ni catalyst demonstrated good stability for 200 h in a fixed-bed reactor without remarkable decrease in the THFA conversion or 1,5–PDO selectivity at half and almost full conversion of THFA (Fig. 1b and Supplementary Fig. 6), which could be attributed to the inhibited agglomeration of metallic Ni species (Supplementary Fig. 7a)^38^ and the enhanced stability in the Ce^3+^ species of CeO_x_ cluster (Supplementary Fig. 7b)^5^.

### Structural characterization of the CeO_x_/Ni catalysts

**Fig. 2:**
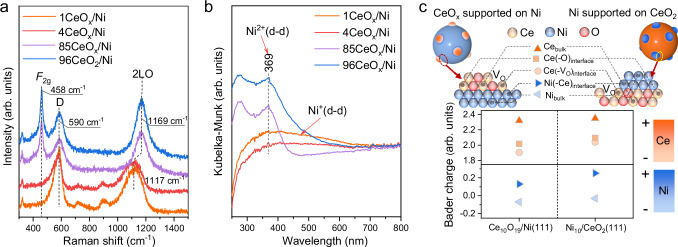
The structural analysis of nCeO_x_/Ni catalysts. **a** UV-Raman (325 nm) and **b** UV-vis adsorption spectra of 1CeO_x_/Ni (orange), 4CeO_x_/Ni (red), 85CeO_x_/Ni (purple), and 96CeO_x_/Ni (blue) catalysts. **c** Bader charge analysis of Ce and Ni species within inverse Ce_10_O_19_/Ni(111) and conventional Ni_10_/CeO_2_(111) catalysts, respectively.

The valence state of surface Ce species was studied by quasi in situ XPS. As shown in Supplementary Fig. 8a, the 4CeO_x_/Ni and 1CeO_x_/Ni catalysts contained 67% and 70% of Ce^3+^ species by calculating the corresponding peak areas of Ce^3+^ and Ce^4+^ species^39^, respectively, which were more than those in the 96CeO_x_/Ni (41%) and 85CeO_x_/Ni (42%) catalysts, indicating that the reduction of Ce^4+^ to Ce^3+^ was promoted over the inverse catalyst. The chemical information of Ce species could also be determined by the ultraviolet Raman spectroscopy (UV-Raman, 325 nm). As shown in Fig. 2a, the *F*_2g_ and D bands at around 458 and 590 cm^−1^ observed over the conventional 96CeO_x_/Ni and 85CeO_x_/Ni catalysts were ascribed to the Ce–O stretch in CeO_2_ lattice and oxygen vacancy related defect, respectively^40^. However, the negligible *F*_2g_ bands for the inverse 4CeO_x_/Ni and 1CeO_x_/Ni catalysts revealed the increase in crystal structure disorder of the CeO_x_ cluster on the inverse catalysts^40^, which could be associated with the weakening of Ce–O bond, since the remarkable red-shift of the second order longitudinal optical mode (2LO) bands was observed from 1169 cm^−1^ for the 96CeO_x_/Ni and 85CeO_x_/Ni catalysts to 1117 cm^−1^ for the 4CeO_x_/Ni and 1CeO_x_/Ni catalysts^41^.

Since the Ni^δ+^ species was hard to identify by the Ni *2p* XPS spectra (Supplementary Fig. 8b)^42^, the electronic state of surface Ni species was analyzed by ultraviolet-visible light (UV-vis) adsorption spectroscopy. As shown in Fig. 2b, the strong adsorption bands at 369 nm are assigned to the d–d transition of Ni^2+^ species in the 96CeO_x_/Ni and 85CeO_x_/Ni catalysts^43^. While, a new broadband from 400 to 600 nm appeared over 4CeO_x_/Ni and 1CeO_x_/Ni catalysts with a decrease in the intensity of Ni^2+^ bands, which indicated the remarkable reduction of Ni^2+^ to Ni^δ+^ (0 < δ < 1) species^42,44^. Hydrogen temperature-programmed reduction (H_2_-TPR) experiments were performed to study the interaction between Ni and Ce species on the nCeO_x_/Ni catalysts. As shown in Supplementary Fig. 8c, the peaks at 130 °C for the inverse 4CeO_x_/Ni and 1CeO_x_/Ni catalysts were attributed to the reduction of surface-adsorbed oxygen species associated with the formation of interfacial Ni–V_O_–Ce^45^. In contrast, no remarkable reduction peak between 40 °C and 200 °C was observed over the conventional 85CeO_x_/Ni and 96CeO_x_/Ni catalysts, indicating that the reduction of interfacial Ni–O–Ce species over the conventional catalyst could occur at higher temperature, and the corresponding reduction peak might be overlapped with the broad peak at around 320 °C belonged to the reduction of NiO to metallic Ni^46^. The low reduction temperature of Ni–V_O_–Ce species on the 4CeO_x_/Ni and 1CeO_x_/Ni catalysts indicated the strong interaction between interfacial Ni and Ce species on inverse catalyst^47^, which could be further demonstrated by quasi in situ electron paramagnetic resonance (EPR) spectroscopy, where the *g*-value of Ni species shifted from 2.39 in the conventional 96CeO_x_/Ni catalysts to 2.16 in the inverse 4CeO_x_/Ni catalysts (Supplementary Fig. 8d)^42,48^.

As indicated in the above catalyst screening tests, the interface catalytic sites containing Ni and Ce species were responsible for the enhanced hydrogenolysis of THFA in terms of catalytic activity and stability, particularly under solvent-free condition. Therefore, the structure of interface Ni-Ce was explored by using theoretical calculations with the models of Ce_10_O_19_/Ni(111) and Ni_10_/CeO_2_(111) representing the inverse and conventional catalyst, respectively^49,50^. Based on the difference in the coordination structure of Ce species, it could be categorized into three Ce species (Fig. 2c): Ce in the bulk CeO_2_ (labeled as Ce_bulk_), interfacial Ce without adjacent oxygen vacancy (labeled as Ce(–O)_interface_), and interfacial Ce associated with an oxygen vacancy (labeled as Ce(–V_O_)_interface_). The corresponding Ni species were denoted as Ni_bulk_ and Ni(–Ce)_interface_, where the latter was defined as the interfacial Ni adjacent to the Ce. Bader charge analysis demonstrated that the presence of oxygen vacancy could modulate the electronic state^51^, resulting in the lower valence state of Ce(–V_O_)_interface_ species (+2.04) than that of Ce(–O)_interface_ species (+2.09) on the conventional Ni_10_/CeO_2_(111) catalyst (Supplementary Fig. 9). It can be seen that the electronic interaction between the CeO_x_ cluster and bulk Ni was significantly enhanced on the inverse Ce_10_O_19_/Ni(111) catalyst. Therefore, a larger electron density of Ce(–V_O_)_interface_ species (charge of +1.90) was generated. Simultaneously, the strong interaction also led to a lower Bader charge of Ni(-Ce)_interface_ species on the inverse catalyst at +0.13 compared to that on the conventional catalyst at +0.25 (Supplementary Fig. 10). The results manifested that the electron transfer from the metallic Ni support to the interfacial Ce and Ni species was enhanced on the inverse oxide/metal catalyst due to the strong electronic interaction, which was supposed to enhance the catalytic activity of ring-opening hydrogenation of THFA into 1,5–PDO.

### Investigations on the selective adsorption of oxygenate groups on CeO_x_/Ni calalysts

**Fig. 3:**
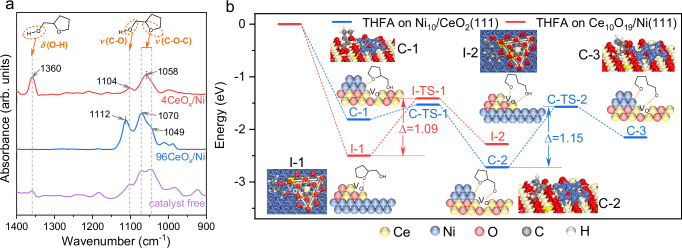
Selective adsorption and activation of oxygenic groups on catalyst surface. **a** In situ FT-IR spectra analysis of THFA (purple) and THFA adsorbed on 4CeO_x_/Ni (red) and 96CeO_x_/Ni (blue) catalysts. **b** DFT calculations for the selective adsorption of THFA and subsequent cleavage of etheric C2–O bond over Ce_10_O_19_/Ni(111) (red) and Ni_10_/CeO_2_(111) (blue), respectively. Atom colors: Ce (yellow), Ni (blue), C (gray), O (red), H (white).

Since the interfacial electronic effect could control the selective adsorption of substrate or intermediate^52^, it is herein supposed that the strong electronic interaction over the inverse catalysts could tune the adsorption mode of THFA. After THFA saturated adsorption at 50 °C, a single adsorption peak at 1070 cm^−1^ with a shoulder peak at 1049 cm^−1^ for the 96CeO_x_/Ni catalyst was detected using in situ FT-IR and was assignable to be the etheric C–O–C bond vibration referred to the corresponding adsorption peak of free THFA (Fig. 3a)^53^. While, a peak shift from 1070 cm^−1^ to 1058 cm^−1^ was observed over the 4CeO_x_/Ni catalyst, indicating the difference in the adsorption mode of etheric C–O bond between the inverse catalyst and the conventional 96CeO_x_/Ni catalyst. The adsorption peaks of C–O bond and O–H bond in the hydroxyl group on the 4CeO_x_/Ni catalyst were located at 1104 cm^−1^ and 1360 cm^−1^, which are the same as those of free THFA^54,55^. However, when THFA was adsorbed on the 96CeO_x_/Ni catalyst, the vibration peak of the O–H bond disappeared, revealing the cleavage of O–H bond, and simultaneously the adsorption peak of the hydroxyl C–O bond blue-shifted to 1112 cm^−1^ suggesting the strong adsorption of the corresponding deprotonated hydroxyl oxygen which was supposed to locate at the oxygen vacancy sites on the conventional catalyst^56,57^.

The selective adsorption of THFA was further studied by the DFT calculation. The THFA molecule was tried to adsorb at different interfacial sites on the inverse Ce_10_O_19_/Ni(111) catalyst (Supplementary Fig. 11), and it was found that THFA preferred to adsorb at the interface Ce sites due to the highest adsorption energy of −2.50 eV, where the etheric oxygen atom of THFA was bonded with the electron-rich Ce(–V_O_)_interface_ atom (I–1 in Fig. 3b and Supplementary Fig. 11a) rather than Ce(–O)_interface_ atom (Supplementary Fig. 11c). However, on the conventional Ni_10_/CeO_2_(111) catalyst, the etheric oxygen atom adsorbed at the Ni(–Ce)_interface_ site with a higher adsorption energy of –1.81 eV (C–1 in Fig. 3b and Supplementary Fig. 12a). The adsorbed THFA molecule on the Ni_10_/CeO_2_(111) catalyst was unstable and proceeded a deprotonation process by overcoming a low barrier of 0.28 eV (C–1→C–2 in Fig. 3b and Supplementary Fig. 13), which afforded a stable intermediate through strong bonding with oxygen vacancy. The different adsorption modes of THFA could be further demonstrated by quasi in situ XPS adsorption experiment. The relative content of oxygen vacancy (determined by the ratio of Ce^3+^/(Ce^3+^+Ce^4+^)) and the surface-adsorbed O species associated with oxygen vacancy (denoted as O_II_, calculated by its corresponding area ratio to lattice oxygen of O_I_) were monitored, respectively^35,58^. As shown in Supplementary Fig. 14, the relative content of O_II_ species increased from 1.6 to 2.9 with no remarkable change of oxygen vacancy after THFA adsorption over the inverse 4CeO_x_/Ni catalyst. By contrast, the THFA adsorbed on conventional 96CeO_x_/Ni catalyst led to the decrease in O_II_ content from 0.6 to 0.5 with the diminishment of oxygen vacancy content (Ce^3+^/(Ce^3+^+Ce^4+^) ratio from 41% to 34%). These results manifested the adsorption mode of THFA over inverse 4CeO_x_/Ni catalyst via selective adsorption of etheric oxygen at the Ce(–V_O_)_interface_ site adjacent to oxygen vacancy, while both the adsorption of etheric oxygen at the Ni(–Ce)_interface_ site and deprotonation of hydroxyl at the oxygen vacancy occurred simultaneously over conventional 96CeO_x_/Ni catalyst.

The adsorption mode of THFA significantly affects the catalytic activity of etheric C–O bond cleavage which is the key step in the ring-opening hydrogenation of THFA. When the THFA was adsorbed on the Ce_10_O_19_/Ni(111) catalyst, the adsorbed intermediate of I–1 underwent ring-opening via C2–O bond cleavage by overcoming a barrier of 1.09 eV (I–1→I–2 in Fig. 3b and Supplementary Fig. 15). However, a higher barrier of 1.15 eV was necessary for the cleavage of C2–O bond in the deprotonated intermediate of C–2 over the Ni_10_/CeO_2_(111) catalyst (C–2→C–3 in Fig. 3b and Supplementary Fig. 13), which gave rise to the higher catalytic activity by inverse 4CeO_x_/Ni catalyst than that of conventional 96CeO_x_/Ni catalyst in the ring-opening hydrogenation of THFA into 1,5–PDO.

### Investigations on the adsorption and activation of hydrogen species on CeO_x_/Ni catalysts

**Fig. 4:**
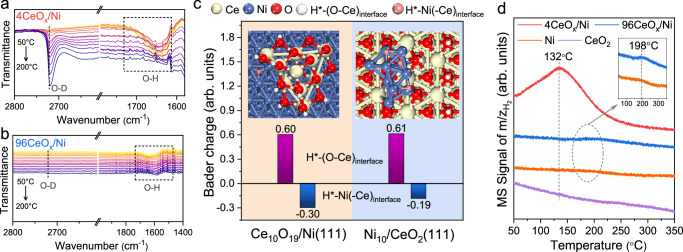
Quantitative analysis of hydrogen species on catalyst surface. The in situ FT-IR spectra for the analysis of surface hydrogen species in a D_2_ flow: **a** inverse 4CeO_x_/Ni catalyst and **b** conventional 96CeO_x_/Ni catalyst. **c** Bader charge analysis of H species on Ce_10_O_19_/Ni(111) and Ni_10_/CeO_2_(111). Atom colors: Ce (yellow), Ni (blue), O (red), H (white and pink). **d** H_2_-TPD-MS profiles over 4CeO_x_/Ni (red line), 96CeO_x_/Ni (blue line), Ni (orange line), and CeO_2_ (purple line).

The activated H species (i.e., H•, H^–^) via homolytic or heterolytic dissociation of H_2_ have a profound influence on the catalytic hydrogenation activity^59^. Before measurements by in situ FT-IR spectroscopy to study the formation of hydride species on the catalysts, the catalysts were pre-reduced by H_2_. As shown in Fig. 4a, the O–H vibration bands between 1600–1720 cm^–1^ were observed over the inverse 4CeO_x_/Ni catalyst^60^. As the temperature increased from 50 °C to 200 °C under the D_2_ atmosphere, the intensity of O–H peak gradually diminished, while the O–D stretching vibration peak appeared at 2720 cm^–1^ when the temperature reached 100 °C, and the peak intensity increased with raising temperature, indicating the exchange reaction between H^δ+^ and D^δ+^ in surface hydroxyl group occurred on the inverse 4CeO_x_/Ni catalyst^61^. However, no remarkable H^δ+^–D^δ+^ exchange reaction proceeded on the 96CeO_x_/Ni conventional catalyst, since the negligible O–D vibration peak was observed in Fig. 4b. It was worth noting that both D_2_ and H_2_ could be readily activated and transformed into HD via H–D exchange reaction on the conventional 96CeO_x_/Ni catalyst according to the obvious HD signal detected below 100 °C (Supplementary Fig. 16). The results manifested that H atom instead of H^δ+^ species was formed on the conventional 96CeO_x_/Ni catalyst via homolytic cleavage of H_2_. In contrast, the formation of H^δ+^ species suggested the heterolytic dissociation of H_2_ on the inverse 4CeO_x_/Ni catalyst, which simultaneously produced the corresponding H^δ–^ species.

To study the location of hydrogen species on the inverse and conventional catalysts, the DFT calculations were conducted. As shown in Fig. 4c, the H species preferred to bond with the interfacial Ni atoms (denoted as H*–Ni(-Ce)_interface_) and O atoms of CeO_x_ (denoted as H*–(O–Ce)_interface_) rather than with the interfacial Ce atom. Further, Bader charge analysis (Supplementary Fig. 17) shows that the valence state of H*–Ni(-Ce)_interface_ species (–0.30 a.u.) on Ce_10_O_19_/Ni(111) was more negative than that (–0.19 a.u.) on Ni_10_/CeO_2_(111), indicating that H^δ–^ species tend to form on the inverse catalyst due to bonding with the electron-rich Ni(-Ce)_interface_. Combining with the above structural analysis, the facile formation of H^δ–^ species over inverse catalyst could be attributed to the strong electronic interaction which boosted the charge transfer from bulk Ni to the interfacial Ni.

It is recognized that the adsorbed H species can undergo recombination and desorb as H_2_ molecule from the catalyst surface at an appropriate temperature. Here, an obvious H_2_ desorption peak at 132 °C for inverse 4CeO_x_/Ni catalyst was observed by hydrogen temperature-programmed desorption combined with mass spectrometry (H_2_-TPD-MS, red line in Fig. 4d), which was attributed to the recombination between adsorbed H^δ+^ and H^δ–^ species^62^. While, the 96CeO_x_/Ni catalyst (blue line) desorbed H_2_ at a higher temperature (198 °C) similar to that of metal Ni catalyst (orange line), indicating the desorption of atomic state H on the conventional catalyst^63^. Their relative content of adsorbed H species on 4CeO_x_/Ni and 96CeO_x_/Ni catalysts were calculated by area integrating of the corresponding peaks to be 9.64 and 0.77 μmol_H2_ g_cat_^–1^, respectively. The results were consistent with the great difference in their THFA hydrogenolysis activity as shown in Fig. 1a. The kinetic analysis of H_2_ pressure further demonstrated the correlation of reactive activity with the content of H species, which could be tuned by changing H_2_ pressure^24^. As shown in Supplementary Fig. 18, the specific rate of 1,5–PDO increased as the pressure increased from 0.4 MPa to 4.0 MPa. Further increase in H_2_ pressure led to the saturated adsorption of H species on the 4CeO_x_/Ni catalyst, and no significant improvement in catalytic activity over 4.0 MPa was observed. Therefore, the superior hydrogenolysis activity of the inverse catalyst could also be associated with the high content of H^δ−^ species on 4CeO_x_/Ni, since the H^δ−^ species was reported to prefer to reduce the polar group^64^.

### Selective hydrogenation transformation of representative biomass-derived molecules and polyether

**Fig. 5:**
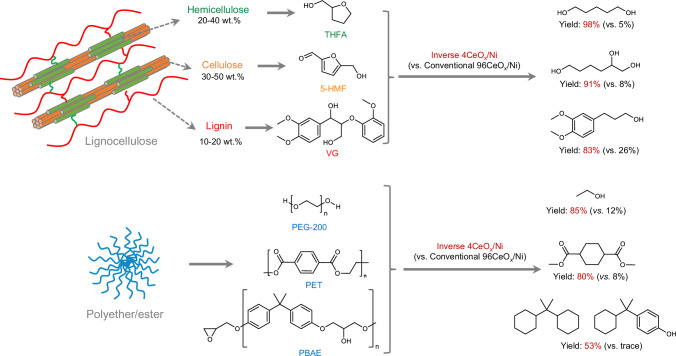
Transformation of representative lignocellulose derivates and polyether/ester over inverse 4CeO_x_/Ni catalyst. THFA tetrahydrofurfuryl alcohol, 5–HMF 5–hydroxymethyl furfural, VG veratrylglycerol-β-guaiacyl ether, PEG polyethylene glycol, PET polyethylene terephthalate, PBAE poly(bisphenol A-co-epichlorohydrin) glycidyl end-capped.

In addition to the ring-opening hydrogenolysis of THFA derived from hemicellulose (Fig. 5, Supplementary Table 5), the development of a general and efficient catalyst towards etheric C–O bond hydrogenolysis of other lignocellulose-derived molecules and even oxygen-containing plastic waste into valuable hydroxyl compounds was highly desirable and challenging. To our delight, 5–hydroxymethyl furfural (5–HMF), a platform compound readily obtained from cellulose^65^, yielded 91% of 1,2,6–hexanetriol through the hydrogenolysis cleavage of furanic C–O bond on inverse 4CeO_x_/Ni catalyst, while its conventional counterpart only gave 1,2,6–hexanetriol in 8% yield. For the selective cleavage of the C–O bonds of veratrylglycerol-β-guaiacyl ether (VG), a representative moiety of lignin molecule containing β–O–4 linkages (43–65% in lignin)^66^, 4CeO_x_/Ni catalyst also demonstrated high activity (83% yield) in the hydrogenolysis of VG to produce the corresponding 3–(3,4–dimethoxyphenyl)–1–propanol product by cleavage of the etheric β–O–4 bond and the neighboring internal hydroxyl C–O bond, which was much higher than that of 26% yield over conventional 96CeO_x_/Ni catalyst. Moreover, this 4CeO_x_/Ni-catalyzed reductive scission of etheric C–O bond using H_2_ was also found to be a powerful strategy to depolymerize polyether. Using polyethylene glycol (PEG–200) as the model polyether, the corresponding depolymerization product, ethanol, was obtained in 100% atom-efficiency with up to 85% yield under similar reaction conditions with the hydrogenolysis of bio-derived molecules. 4CeO_x_/Ni also depolymerized the poly(bisphenol A-co-epichlorohydrin) glycidyl end-capped (PBAE) via selective hydrogenolysis of etheric C–O bond, achieving a 53% yield of propane–2,2–diyldicyclohexane and 4–(2–cyclohexylpropan–2–yl)phenol. In addition, the 4CeO_x_/Ni catalyst demonstrated high catalytic activity in the hydrogenation conversion of polyethylene terephthalate (PET) to dimethyl cyclohexane dicarboxylate in 80% yield. These results show the great potential of such inverse 4CeO_x_/Ni catalyst in the recycle of plastics waste.

## Discussion

In summary, the inverse catalyst of 4CeO_x_/Ni demonstrated a remarkable enhancement in catalytic activity and selectivity for hydrogenolysis of various etheric C–O bonds in lignocellulose derivatives and polyether to produce valuable hydroxyl compounds with an atom-economic manner. Furthermore, the stability of this non-precious metal catalyst was well verified in the ring-opening hydrogenation of THFA to 1,5–PDO in a fixed-bed reactor with unprecedented activity under solvent-free condition. The systematic experimental and computational studies revealed that the obvious advantage of inverse 4CeO_x_/Ni catalyst over the conventional 96CeO_x_/Ni catalyst was enabled by the strong interfacial electronic interaction between the oxide and metal, which promoted the cleavage of etheric C–O bond through the enhanced adsorption of etheric oxygen adsorbed at the electron-rich interfacial Ce sites adjacent to oxygen vacancy, and dominated the formation of high content of H^δ−^ species adsorbed on interfacial Ni sites over the inverse catalyst, both cooperatively boosted the reaction rate of C–O bond hydrogenolysis. This work advanced the fundamental understanding of interfacial electronic interaction over oxide/metal inverse catalyst and provided a promising catalyst design strategy for the efficient transformation of biomass and oxygen-containing polymer waste.

## Methods

### Chemicals

Nickel nitrate hexahydrate (Ni(NO_3_)_2_·6H_2_O, 99%), cobalt nitrate hexahydrate (Co(NO_3_)_2_·6H_2_O, 99%), copper nitrate trihydrate (Cu(NO_3_)_2_·3H_2_O, 99%), cerium nitrate hexahydrate (Ce(NO_3_)_3_·6H_2_O, 99.9%), ammonium perrhenate (NH_4_ReO_4_, 99.9%), ammonium metavanadate (NH_4_VO_3_, 99%), ammonium paratungstate ((NH_4_)_10_H_2_(W_2_O_7_)_6_, >99%), ammonium molybdate tetrahydrate ((NH_4_)_6_Mo_7_O_24_·4H_2_O, >99%), oxalic acid (99%), tetrahydrofurfuryl alcohol (THFA, 99%), 5–hydroxymethyl furfural (97%), veratrylglycerol-β-guaiacyl ether (97%), polyethylene glycol (PEG, M_V_ = 200), PET (white granular, intrinsic viscosity of 0.82 dL/g), and PBAE (Mn ≈ 1075) were purchased from Adamas Reagent Ltd. All the chemicals and reagents were used as received without further purification, and the corresponding aqueous solutions were prepared with DI water (18.25 MΩ cm).

### Preparation of nCeO_x_/Ni catalysts

A series of Ni-supported CeO_2_ catalysts with different CeO_2_ loading (denoted as nCeO_x_/Ni, *n* = 1, 4, 85, and 96) were prepared by the coprecipitation method. Typically, for the 4CeO_2_/Ni catalyst, 200 mL of aqueous solution containing 23.2 g of Ni(NO_3_)_2_·6H_2_O and 0.43 g of Ce(NO_3_)_3_·6H_2_O was added dropwise into 200 mL of the oxalic acid solution (0.8 mol L^−1^) under vigorous stirring. After being continuously stirred for one more hour, the mixture was sealed in a 500 mL Teflon bottle and hydrothermally treated in a stainless-steel autoclave at 140 °C for 24 h. The filter cake was washed with DI water three times. Then, the dried sample was calcined at 400 °C for 3 h in the air. The obtained powder was denoted as the calcined catalyst. Before the reaction, the calcined catalyst was reduced in a H_2_/N_2_ flow (10 vol %) at 350 °C for 3 h with a controlled heating rate of 2 °C min^–1^ in the fixed-bed reactor. For the preparation of 96CeO_x_/Ni catalyst, 1.53 g of Ni(NO_3_)_2_·6H_2_O and 21.7 g of Ce(NO_3_)_3_·6H_2_O were used as the precursors. Similarly, for the 1CeO_x_/Ni catalyst, 23.2 g of Ni(NO_3_)_2_·6H_2_O and 0.11 g of Ce(NO_3_)_3_·6H_2_O were used. The 85CeO_x_/Ni catalyst was prepared by precipitating 5.81 g of Ni(NO_3_)_2_·6H_2_O and 17.4 g of Ce(NO_3_)_3_·6H_2_O with oxalic acid.

### Preparation of physical mixed PM-4CeO_2_ + Ni catalyst

For the preparation of the physical mixed catalyst of PM-4CeO_2_ + Ni, CeO_2_ and NiO powders were first prepared by the coprecipitation method, using Ce(NO_3_)_3_·6H_2_O (23.2 g) and Ni(NO_3_)_2_·6H_2_O (23.3 g) as precursors and oxalic acid as the precipitant. After drying and calcination, the resulting CeO_2_ (0.29 g) and NiO (10.0 g) were physically mixed in a molar ratio of 1/80 (Ce to Ni) using an agate mortar. Finally, the mixture was reduced by H_2_/N_2_ in a fixed-bed reactor before the hydrogenation reaction.

### Preparation of IM-M’O_x_/Ni catalysts

The catalysts of IM-M’O_x_/Ni (M’ = Ce, Re, Mo, V, and W) were prepared by the impregnation method (IM). The corresponding precursors, such as Ce(NO_3_)_3_·6H_2_O (0.073 g), NH_4_ReO_4_ (0.045 g), NH_4_VO_3_ (0.019 g), (NH_4_)_10_H_2_(W_2_O_7_)_6_ (0.043 g), (NH_4_)_6_Mo_7_O_24_·4H_2_O (0.029 g), were respectively loaded on the prepared NiO (1.0 g) with the molar ratio of M’ to Ni of 1/80. After calcined at 400 °C and subsequently reduced at 350 °C, the catalysts of IM-M’O_x_/Ni were obtained.

### Structural characterization

High-resolution transmission electron microscopy images, high-angle annular dark field scanning transmission electron microscopy images, and the energy dispersive X-ray spectroscopy mapping were obtained using an FEI F20 (200 kV). XRD experiments were performed on a Rigaku SmartLab diffractometer with Cu-Kα radiation. Quasi in situ XPS experiments were performed on a Thermo Scientific NEXSA Instrument. Before the XPS measurements, the catalyst was reduced in a glass tube, which was then sealed and transferred into a glove box without exposure to air. Subsequently, the sample was loaded on a sealed specimen stage and transferred into the XPS analysis chamber. All of the operations were carried out at room temperature inside a glove box. UV-Raman spectra were collected on a Labram HR Evolution (HORIBA) with a semiconductor laser (325 nm, 1 mW). The diffuse reflection ultraviolet-visible spectra were collected on a UV-vis spectrophotometer (Shimadzu, UV-2700). Quasi in situ EPR experiments were carried out on a JEOL RESONANCE JES-X320 spectrometer operating at X-band frequency (*v* ≈ 9.15 GHz) at 25 °C. Before the EPR measurements, the catalyst powder was reduced in a glass tube, which was then sealed and transferred into a glove box without exposure to air. Subsequently, the sample was sealed in a glass capillary under a N_2_ atmosphere. All of the operations were performed at room temperature inside a glove box. The metal content was determined by ICP-MS on a Thermo Fisher ICAP RQ instrument. The nitrogen adsorption-desorption isotherms were obtained on an ASAP 2020 Micromeritics instrument at 77 K, and the specific surface area was calculated using Brunauer-Emmett-Teller equation.

### In situ infrared Fourier transform measurements

The adsorption configuration of THFA on the catalysts was studied using in situ FT-IR experiments. The experiments were conducted on a Bruker infrared spectrometer (Vertex 70). Before measurements, the calcined catalyst was first reduced at 350 °C and then cooled to 50 °C in a 10 vol % H_2_/N_2_ flow. In the THFA hydrogenolysis process, the chamber was first evacuated to a vacuum. Then, saturated THFA gas was introduced into the chamber for 1 h. After the saturated adsorption of THFA on the catalyst surface, a 10 vol % H_2_/N_2_ flow (30 mL min^–1^) was purged for 1 h to remove the physically adsorbed THFA molecule., and the corresponding IR spectra were collected. In the H–D exchange experiments, the catalyst was pre-reduced at 350 °C in a H_2_/N_2_ flow. After cooling to 50 °C under the Ar atmosphere, the chamber was purged by D_2_ for 0.5 h. Subsequently, the sample was ramped up to 200 °C in a D_2_ flow, and the corresponding IR spectra were monitored.

### Quasi in situ XPS spectra analysis for THFA adsorption analysis

Firstly, the reduced catalyst was transferred into a glove box without exposure to air. Then, the catalyst sample was impregnated in THFA for several minutes. After saturation adsorption of THFA, the catalyst was vacuumed to remove the physically adsorbed THFA. Subsequently, the catalyst was transferred into the XPS analysis chamber.

### H–D exchange experiments

First, the calcined catalyst was reduced at 350 °C in H_2_/N_2_ flow (30 mL min^–1^, 10 vol % H_2_) and then cooled to 50 °C. The chamber was purged by D_2_ (15 mL min^–1^) mixed with H_2_ (15 mL min^–1^) for 1 h. Subsequently, the sample was heated to 400 °C with a heating rate of 10 °C min^–1^ in a mixture gas of H_2_ + D_2_, and the MS signal of HD (m/z = 3) was collected and analyzed with a mass spectrometer. The formation rate of HD was evaluated by the corresponding ion current intensity.

### Hydrogen temperature-programmed desorption

H_2_-TPD experiments were performed on a TP5080 chemisorption analyzer (Tianjin Xianquan Co., Ltd, China) equipped with a mass spectrometry detector. Prior to H_2_ desorption, the calcined catalysts were pretreated at 350 °C and then cooled down to 50 °C using a H_2_/N_2_ flow (30 mL min^–1^, 10 vol % H_2_). After the cooling process, the sample was purged for 1 h and then ramped up to 400 °C at a heating rate of 10 °C min^–1^ in a He flow (30 mL min^–1^). During the ramping process, the MS signals of H_2_ (m/z = 2) were recorded.

### Carbon monoxide temperature-programmed desorption

CO-TPD was performed on a chemisorption analyzer equipped with a mass spectrometry detector. Before measurements, the catalysts were pre-reduced at 350 °C using a 10 vol % H_2_/N_2_, and then CO molecules were adsorbed at 30 °C for 0.5 h in a flow of 10 vol % CO/N_2_. After purging the physically adsorbed CO molecules, the sample was heated at 10 °C min^–1^ to 800 °C in Ar, and the MS signals of CO (m/z = 28) and CO_2_ (m/z = 44) were recorded.

### Hydrogen temperature-programmed reduction

H_2_-TPR was carried out with a chemisorption analyzer equipped with a thermal conductivity detector (TCD). Firstly, the catalysts were pretreated at 400 °C for 1 h under He flow. After cooling down to 30 °C, the quartz tube reactor was purged with flowing H_2_/N_2_ (10 vol % H_2_). The sample was ramped to 800 °C with a heating rate of 10 °C min^–1^ in 10 vol % H_2_/N_2_ flow (30 mL min^-1^), and the TCD signal was recorded.

### Hydrogenolysis of THFA in a fixed-bed reactor

The catalytic activity of the nCeO_x_/Ni catalyst was evaluated in a fixed-bed reactor. Typically, 5.4 g of calcined 4CeO_x_/Ni catalyst was formed into 40–60 mesh pellets. The pellets were then loaded into the stainless-steel tubular reactor with quartz liner (inner diameter of 10 mm). Before the reaction, the calcined catalyst was reduced at 350 °C using a 10 vol % H_2_/N_2_ flow (40 mL min^–1^) for 3 h. After cooling down to the desired temperature, a feed of THFA (solvent-free, 0.02 mL min^−1^) was pumped into the reactor, and the hydrogenolysis reaction was carried out at a temperature of 160 °C under flowing H_2_ (4 MPa, 50 mL min^–1^). During the reaction, the product was collected through a condenser pipe at –10 °C. The collected products were quantitatively analyzed using gas chromatography (GC) equipped with an FFAP chromatographic column and flame ionization detector (Shimadzu GC-2014C). The specific reaction rate was calculated using the following equation:

$$r=\frac{{molar\; yield\; of} \, 1,5-{PDO} \, (\mu {mol})}{{mass\; of\; catalyst}\left(g\right)\times {reaction\; time}(\min )}$$

Here, the molar yield of 1,5–PDO was determined from the conversion of THFA and the selectivity of 1,5–PDO based on three independent measurements.

### Hydrogenation of the biomass compounds and polyether/ester in a batch reactor

For the reaction in a batch reactor, the catalyst was initially reduced at 350 °C for 3 h in a 10 vol % H_2_/N_2_ flow (50 mL min^–1^) and subsequently passivated in a 5 vol% O_2_/N_2_ flow (40 mL min^–1^) at 30 °C for 5 mins. Prior to the reaction, the autoclave was purged three times and then pressured with H_2_ gas. The hydrogenolysis reaction of THFA (0.5 g) was catalyzed by a 0.25 g catalyst in a stainless-steel autoclave (100 mL). The reaction was conducted at a temperature of 160 °C under continuous stirring. The conversion of 5–hydroxymethyl furfural (0.5 g) was catalyzed by catalyst (0.25 g) under similar reaction conditions with the hydrogenolysis of THFA. For the transformation of veratrylglycerol-β-guaiacyl ether (0.25 g), the reaction (0.12 g of catalyst) was carried out at 150 °C. The depolymerization of polyethylene glycol (PEG–200, 0.5 g) was performed by using tetrahydrofuran as the solvent (0.4 g catalyst, 160 °C, 4.0 MPa H_2_). The reaction of 0.5 g of PET (white granular, intrinsic viscosity of 0.82 dL/g) was carried out in methanol under 170 °C (0.4 g catalyst, 4.0 MPa H_2_). The conversion of PBAE (Mn ≈ 1075) was carried out in methanol (0.4 g catalyst, 170 °C, 4.0 MPa H_2_).The quantitative analysis of the obtained products was determined by GC.

### DFT calculation

First-principles calculations were performed using periodic DFT^67^. The spin-polarized generalized gradient approximation with the PBE functional was used to treat exchange-correlation effects. A plane wave basis set with a cutoff energy of 450 eV was selected to describe the valence electrons. The electron-ion interactions were described by the projector augmented wave method^68^^.^ The Brillouin zone integration was performed with a 2 × 2 × 1 Monkhorst-Pack k-mesh. The SCF, the force convergence criteria for structural optimization and the convergence criteria of transition states were set to 1 × 10^–5^ eV, 0.01 eV/Å and 0.03 eV/Å, respectively. The climbing image nudged elastic band and dimer methods were used to optimize the transition state structures. To describe the strongly correlated f-electrons of Ce, we adopt Dudarev’s DFT + U scheme with *U* = 5 eV, following previous research^69^. We used Grimme’s DFT-D3 scheme to include the van der Waals interactions semi-empirically^70^. The optimized conventional cell of ceria with a lattice parameter of 3.871 Å was adopted to construct the oxygen-terminated CeO_2_(111) surface. A (3 × 2√3) supercell with four O–Ce–O trilayers and 15 Å vacuum slab was used to describe the ceria support of the conventional catalyst during the catalytic process. An oxygen atom on the topmost layer of CeO_2_(111) is removed to create oxygen vacancy. A Ni_10_ cluster is adopted to represent the Ni particle, which occupies six surface oxygen sites after optimization. The inverse catalyst is modelled with a four-layer (6 × 6) Ni(111) slab supporting a Ce_10_O_19_ cluster. Considering the large size of the (6 × 6) Ni(111), only Γ point was used in the Brillouin zone sampling.

## Supplementary information

Supplementary Information

Peer Review File

## Source data

Source Data

## Data Availability

All data were available in the main text or the supplementasry materials. Source data of the figures are provided. Source data are provided with this paper.

## References

[1] van Deelen, T. W., Hernández, Mejía, C. & de Jong, K. P. Control of metal-support interactions in heterogeneous catalysts to enhance activity and selectivity. *Nat. Catal.* **2**, 955–970 (2019).

[2] Xu, M. et al. Renaissance of strong metal-support interactions. *J. Am. Chem. Soc.* **146**, 2290–2307 (2024). 38236140 10.1021/jacs.3c09102

[3] Wu, C. et al. Inverse ZrO_2_/Cu as a highly efficient methanol synthesis catalyst from CO_2_ hydrogenation. *Nat. Commun.* **11**, 5767 (2020). 33188189 10.1038/s41467-020-19634-8 PMC7666171

[4] Lyu, Z. et al. Amplified interfacial effect on atomically dispersed RuO_x_-on-Pd 2D inverse nanocatalysts for high-performance oxygen reduction. *Angew. Chem. Int. Ed.* **60**, 16093–16100 (2021). 10.1002/anie.202104013 33884729

[5] Rodriguez, J. A. et al. Inverse oxide/metal catalysts in fundamental studies and practical applications: a perspective of recent developments. *J. Phys. Chem. Lett.* **7**, 2627–2639 (2016). 27327114 10.1021/acs.jpclett.6b00499

[6] Kattel, S., Ramírez, P. J., Chen, J. G., Rodriguez, J. A. & Liu, P. Active sites for CO_2_ hydrogenation to methanol on Cu/ZnO catalysts. *Science* **355**, 1296–1299 (2017). 28336665 10.1126/science.aal3573

[7] Fu, J. et al. Modulating the dynamics of Brønsted acid sites on PtWO_x_ inverse catalyst. *Nat. Catal.* **5**, 144–153 (2022).

[8] Zhang, C. & Wang, F. Catalytic lignin depolymerization to aromatic chemicals. *Acc. Chem. Res.* **53**, 470–484 (2020). 31999099 10.1021/acs.accounts.9b00573

[9] Yun, Y. S., Berdugo-Díaz, C. E. & Flaherty, D. W. Advances in understanding the selective hydrogenolysis of biomass derivatives. *ACS Catal.* **11**, 11193–11232 (2021).

[10] Xu, C., Paone, E., Rodriguez-Padron, D., Luque, R. & Mauriello, F. Recent catalytic routes for the preparation and the upgrading of biomass derived furfural and 5–hydroxymethylfurfural. *Chem. Soc. Rev.* **49**, 4273–4306 (2020). 32453311 10.1039/d0cs00041h

[11] Jing, Y., Guo, Y., Xia, Q., Liu, X. & Wang, Y. Catalytic production of value-added chemicals and liquid fuels from lignocellulosic biomass. *Chem* **5**, 2520–2546 (2019).

[12] Zhao, Z. et al. Synergistic catalysis for promoting ring-opening hydrogenation of biomass-derived cyclic oxygenates. *ACS Catal.* **13**, 5170–5193 (2023).

[13] Hou, Q. et al. Upcycling and catalytic degradation of plastic wastes. *Cell Rep. Phys. Sci.* **2**, 100514 (2021).

[14] He, M., Sun, Y. & Han, B. Green carbon science: efficient carbon resource processing, utilization, and recycling towards carbon neutrality. *Angew. Chem. Int. Ed.* **61**, e202112835 (2022). 10.1002/anie.202112835 34919305

[15] Lee, K., Jing, Y., Wang, Y. & Yan, N. A unified view on catalytic conversion of biomass and waste plastics. *Nat. Rev. Chem.* **6**, 635–652 (2022). 37117711 10.1038/s41570-022-00411-8 PMC9366821

[16] Brentzel, Z. J. et al. Chemicals from biomass: combining ring-opening tautomerization and hydrogenation reactions to produce 1,5–pentanediol from furfural. *ChemSusChem* **10**, 1351–1355 (2017). 28277620 10.1002/cssc.201700178

[17] Huang, K. et al. Conversion of furfural to 1,5–pentanediol: process synthesis and analysis. *ACS Sustain. Chem. Eng.* **5**, 4699–4706 (2017).

[18] Wijaya, H. W., Hara, T., Ichikuni, N. & Shimazu, S. Hydrogenolysis of tetrahydrofurfuryl alcohol to 1,5–pentanediol over a nickel-yttrium oxide catalyst containing ruthenium. *Chem. Lett.* **47**, 103–106 (2018).

[19] Feng, S., Nagao, A., Aihara, T., Miura, H. & Shishido, T. Selective hydrogenolysis of tetrahydrofurfuryl alcohol on Pt/WO_3_/ZrO_2_ catalysts: effect of WO_3_ loading amount on activity. *Catal. Today* **303**, 207–212 (2018).

[20] Pholjaroen, B. et al. Selective hydrogenolysis of tetrahydrofurfuryl alcohol to 1,5–pentanediol over vanadium modified Ir/SiO_2_ catalyst. *Catal. Today* **245**, 93–99 (2015).

[21] Chen, K., Mori, K., Watanabe, H., Nakagawa, Y. & Tomishige, K. C-O bond hydrogenolysis of cyclic ethers with OH groups over rhenium-modified supported iridium catalysts. *J. Catal.* **294**, 171–183 (2012).

[22] Chia, M. et al. Selective hydrogenolysis of polyols and cyclic ethers over bifunctional surface sites on rhodium-rhenium catalysts. *J. Am. Chem. Soc.* **133**, 12675–12689 (2011). 21736345 10.1021/ja2038358

[23] Koso, S. et al. Promoting effect of Mo on the hydrogenolysis of tetrahydrofurfuryl alcohol to 1,5–pentanediol over Rh/SiO_2_. *J. Catal.* **267**, 89–92 (2009). 10.1039/b822942b 19333482

[24] Tomishige, K., Nakagawa, Y. & Tamura, M. Selective hydrogenolysis and hydrogenation using metal catalysts directly modified with metal oxide species. *Green. Chem.* **19**, 2876–2924 (2017).

[25] Guan, J., Peng, G., Cao, Q. & Mu, X. Role of MoO_3_ on a rhodium catalyst in the selective hydrogenolysis of biomass-derived tetrahydrofurfuryl alcohol into 1,5–pentanediol. *J. Phys. Chem. C.* **118**, 25555–25566 (2014).

[26] Sun, D. et al. Production of C4 and C5 alcohols from biomass-derived materials. *Green. Chem.* **18**, 2579–2597 (2016).

[27] Nakagawa, Y. & Tomishige, K. Production of 1,5–pentanediol from biomass via furfural and tetrahydrofurfuryl alcohol. *Catal. Today* **195**, 136–143 (2012).

[28] Wang, Z. et al. Selective hydrogenolysis of tetrahydrofurfuryl alcohol to 1,5–pentanediol over PrO_x_ promoted Ni catalysts. *Catal. Today* **402**, 79–87 (2022).

[29] Al-Yusufi, M. et al. Efficient base nickel-catalyzed hydrogenolysis of furfural-derived tetrahydrofurfuryl alcohol to 1,5–pentanediol. *ACS Sustain. Chem. Eng.* **10**, 4954–4968 (2022).

[30] Soghrati, E., Ong, T. K. C., Poh, C. K., Kawi, S. & Borgna, A. Zeolite–supported nickel phyllosilicate catalyst for C–O hydrogenolysis of cyclic ethers and polyols. *Appl. Catal. B* **235**, 130–142 (2018).

[31] Soghrati, E. et al. C–O hydrogenolysis of tetrahydrofurfuryl alcohol to 1,5–pentanediol over bi-functional nickel-tungsten catalysts. *ChemCatChem* **10**, 4652–4664 (2018).

[32] Lee, J., Xu, Y. & Huber, G. W. High-throughput screening of monometallic catalysts for aqueous-phase hydrogenation of biomass-derived oxygenates. *Appl. Catal. B* **140-141**, 98–107 (2013).

[33] Yan, H. et al. Construction of stabilized bulk-nano interfaces for highly promoted inverse CeO_2_/Cu catalyst. *Nat. Commun.* **10**, 3470 (2019). 31375672 10.1038/s41467-019-11407-2 PMC6677889

[34] Yao, S. et al. One-step conversion of biomass-derived 5-hydroxymethylfurfural to 1,2,6–hexanetriol over Ni–Co–Al mixed oxide catalysts under mild conditions. *ACS Sustain. Chem. Eng.* **2**, 173–180 (2014).

[35] Chen, A. et al. Structure of the catalytically active copper-ceria interfacial perimeter. *Nat. Catal.* **2**, 334–341 (2019).

[36] Gould, T. D. et al. Synthesis of supported Ni catalysts by atomic layer deposition. *J. Catal.* **303**, 9–15 (2013).

[37] Saw, E. T. et al. Bimetallic Ni–Cu catalyst supported on CeO_2_ for high-temperature water–gas shift reaction: methane suppression via enhanced CO adsorption. *J. Catal.* **314**, 32–46 (2014).

[38] Qiu, Z., Guo, X., Mao, J. & Zhou, R. Insights into the structure-performance relationship of CuO_x_-CeO_2_ catalysts for preferential oxidation of CO: Investigation on thermally induced copper migration process. *Appl. Surf. Sci.* **600**, 154100 (2022).

[39] Werner, K. et al. Toward an understanding of selective alkyne hydrogenation on ceria: On the impact of O vacancies on H_2_ interaction with CeO_2_(111). *J. Am. Chem. Soc.* **139**, 17608–17616 (2017). 29131603 10.1021/jacs.7b10021

[40] Sartoretti, E. et al. In situ Raman analyses of the soot oxidation reaction over nanostructured ceria-based catalysts. *Sci. Rep.* **9**, 3875 (2019). 30846727 10.1038/s41598-019-39105-5 PMC6405916

[41] He, L. et al. Cerium-oxide-modified nickel as a non-noble metal catalyst for selective decomposition of hydrous hydrazine to hydrogen. *ACS Catal.* **5**, 1623–1628 (2015).

[42] Srinivas, D., Satyanarayana, C. V. V., Potdar, H. S. & Ratnasamy, P. Structural studies on NiO-CeO_2_-ZrO_2_ catalysts for steam reforming of ethanol. *Appl. Catal. A* **246**, 323–334 (2003).

[43] Espinosa-Alonso, L., de Jong, K. P. & Weckhuysen, B. M. Effect of the nickel precursor on the impregnation and drying of γ-Al_2_O_3_ catalyst bodies: a UV-vis and IR microspectroscopic study. *J. Phys. Chem. C.* **112**, 7201–7209 (2008).

[44] Kambolis, A., Matralis, H., Trovarelli, A. & Papadopoulou, C. Ni/CeO_2_-ZrO_2_ catalysts for the dry reforming of methane. *Appl. Catal. A* **377**, 16–26 (2010).

[45] Liao, X. et al. Highly efficient Ni/CeO_2_ catalyst for the liquid phase hydrogenation of maleic anhydride. *Appl. Catal. A* **488**, 256–264 (2014).

[46] Ang, M. L. et al. Highly active Ni/*x*Na/CeO_2_ catalyst for the water–gas shift reaction: Effect of sodium on methane suppression. *ACS Catal.* **4**, 3237–3248 (2014).

[47] Zhang, S. et al. Towards highly active Pd/CeO_2_ for alkene hydrogenation by tuning Pd dispersion and surface properties of the catalysts. *Nanoscale* **9**, 3140–3149 (2017). 28220171 10.1039/c6nr09297g

[48] Wang, J. Q. et al. Effects of Ni-doping of ceria-based materials on their micro-structures and dynamic oxygen storage and release behaviors. *Catal. Lett.* **140**, 38–48 (2010).

[49] Li, Y., Li, S., Bäumer, M., Ivanova-Shor, E. A. & Moskaleva, L. V. What changes on the inverse catalyst? Insights from CO oxidation on Au-supported ceria nanoparticles using Ab initio molecular dynamics. *ACS Catal.* **10**, 3164–3174 (2020).

[50] Xie, J. et al. Hydrogenolysis of lignin model compounds on Ni nanoparticles surrounding the oxygen vacancy of CeO_2_. *ACS Catal.* **13**, 9577–9587 (2023).

[51] Zhang, S. et al. Solid frustrated-Lewis-pair catalysts constructed by regulations on surface defects of porous nanorods of CeO_2_. *Nat. Commun.* **8**, 15266 (2017). 28516952 10.1038/ncomms15266 PMC5454379

[52] Chen, G. et al. Interfacial electronic effects control the reaction selectivity of platinum catalysts. *Nat. Mater.* **15**, 564–569 (2016). 26808458 10.1038/nmat4555

[53] Zhu, Y. et al. Selective activation of C–OH, C–O–C, or C=C in furfuryl alcohol by engineered Pt sites supported on layered double oxides. *ACS Catal.* **10**, 8032–8041 (2020).

[54] Meng, X. et al. A control over hydrogenation selectivity of furfural via tuning exposed facet of Ni catalysts. *ACS Catal.* **9**, 4226–4235 (2019).

[55] Ding, L. et al. CO_2_ hydrogenation to ethanol over Cu@Na-beta. *Chem* **6**, 2673–2689 (2020).

[56] Fu, J. et al. C–O bond activation using ultralow loading of noble metal catalysts on moderately reducible oxides. *Nat. Catal.* **3**, 446–453 (2020).

[57] Goulas, K. A., Mironenko, A. V., Jenness, G. R., Mazal, T. & Vlachos, D. G. Fundamentals of C–O bond activation on metal oxide catalysts. *Nat. Catal.* **2**, 269–276 (2019).

[58] Zheng, X. et al. Highly efficient porous Fe_x_Ce_1–x_O_2−δ_ with three-dimensional hierarchical nanoflower morphology for H_2_S-selective oxidation. *ACS Catal.* **10**, 3968–3983 (2020).

[59] Zhang, L., Zhou, M., Wang, A. & Zhang, T. Selective hydrogenation over supported metal catalysts: From nanoparticles to single atoms. *Chem. Rev.* **120**, 683–733 (2020). 31549814 10.1021/acs.chemrev.9b00230

[60] Zhou, H. et al. Hydrogenolysis cleavage of the C_sp2_–C_sp3_ bond over a metal-free NbOPO_4_ catalyst. *ACS Catal.* **12**, 4806–4812 (2022).

[61] Qin, R. et al. Alkali ions secure hydrides for catalytic hydrogenation. *Nat. Catal.* **3**, 703–709 (2020).

[62] Li, Z. et al. Oxidation of reduced ceria by incorporation of hydrogen. *Angew. Chem. Int. Ed.* **58**, 14686–14693 (2019). 10.1002/anie.201907117 PMC6790607 31403236

[63] Bartholomew, C. H. Hydrogen adsorption on supported cobalt, iron, and nickel. *Catal. Lett.* **7**, 27–51 (1990).

[64] Mitsudome, T. et al. Design of a silver–cerium dioxide core–shell nanocomposite catalyst for chemoselective reduction reactions. *Angew. Chem. Int. Ed.* **51**, 136–139 (2012). 10.1002/anie.201106244 22025383

[65] Zakrzewska, M. E., Bogel-Łukasik, E. & Bogel-Łukasik, R. Ionic liquid-mediated formation of 5-hydroxymethylfurfural—a promising biomass-derived building block. *Chem. Rev.* **111**, 397–417 (2011). 20973468 10.1021/cr100171a

[66] Li, C., Zhao, X., Wang, A., Huber, G. W. & Zhang, T. Catalytic transformation of lignin for the production of chemicals and fuels. *Chem. Rev.* **115**, 11559–11624 (2015). 26479313 10.1021/acs.chemrev.5b00155

[67] Xi, Y. et al. Mechanistic study of the ceria supported, re-catalyzed deoxydehydration of vicinal OH groups. *Catal. Sci. Technol.* **8**, 5750–5762 (2018).

[68] Kresse, G. & Joubert, D. From ultrasoft pseudopotentials to the projector augmented-wave method. *Phys. Rev. B* **59**, 1758–1775 (1999).

[69] Nolan, M., Grigoleit, S., Sayle, D. C., Parker, S. C. & Watson, G. W. Density functional theory studies of the structure and electronic structure of pure and defective low index surfaces of ceria. *Surf. Sci.* **576**, 217–229 (2005).

[70] Grimme, S., Antony, J., Ehrlich, S. & Krieg, H. A consistent and accurate ab initio parametrization of density functional dispersion correction (DFT-D) for the 94 elements H-Pu. *J. Chem. Phys.* **132**, 154104 (2010). 20423165 10.1063/1.3382344

