# Supplementary material for: Inverse ceria-nickel catalyst for enhanced C–O bond hydrogenolysis of biomass and polyether
Source: Nat Commun. 2024 Sep 30;15:8444. doi: 10.1038/s41467-024-52704-9 (PMC11443077; doi:10.1038/s41467-024-52704-9)
Supplement: Supplementary file 1 — Supplementary Information [file 41467_2024_52704_MOESM1_ESM.pdf]

Supplementary Information

# Inverse ceria-nickel catalyst for enhanced C–O bond hydrogenolysis of biomass and polyether

Zelun Zhao[1], Guang Gao[1], Yongjie Xi[1], Jia Wang[1], Peng Sun[1], Qi Liu[1], Chengyang Li[1], Zhiwei Huang[1]* & Fuwei Li[1,2]*

[1]State Key Laboratory of Low Carbon Catalysis and Carbon Dioxide Utilization, State Key Laboratory for Oxo Synthesis and Selective Oxidation, Lanzhou Institute of Chemical Physics, Chinese Academy of Sciences, Lanzhou 730000, China

[2]School of Chemical Engineering, University of Chinese Academy of Sciences, Beijing 100049, China

These authors contributed equally: Zelun Zhao, Guang Gao, Yongjie Xi.

E-mail: zwhuang@licp.cas.cn, fuweili@ucas.ac.cn

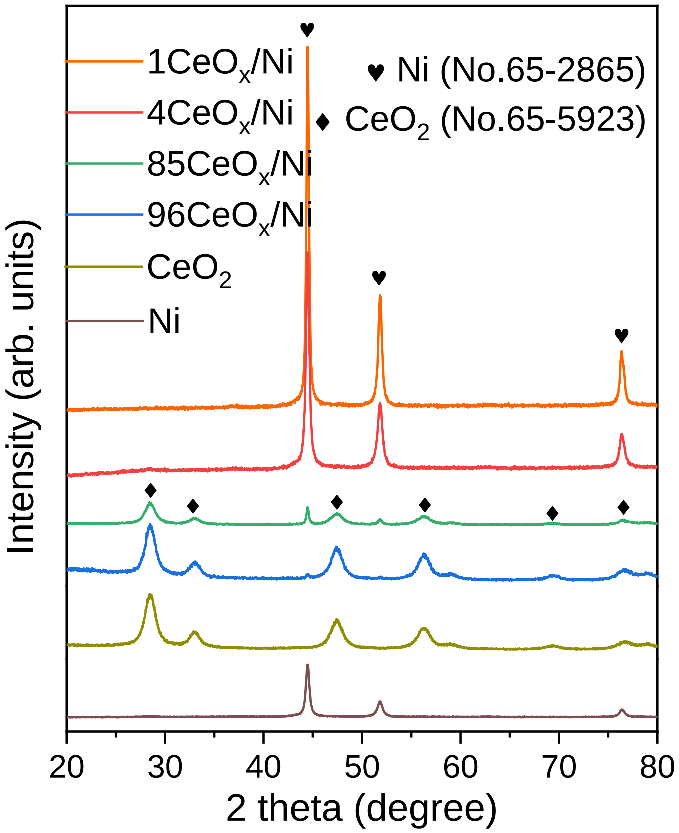


**Supplementary Fig. 1. Structural analysis of catalysts.** The XRD patterns of $1CeO_x/Ni$, $4CeO_x/Ni$, $85CeO_x/Ni$, $96CeO_x/Ni$, $CeO_2$, and Ni samples.

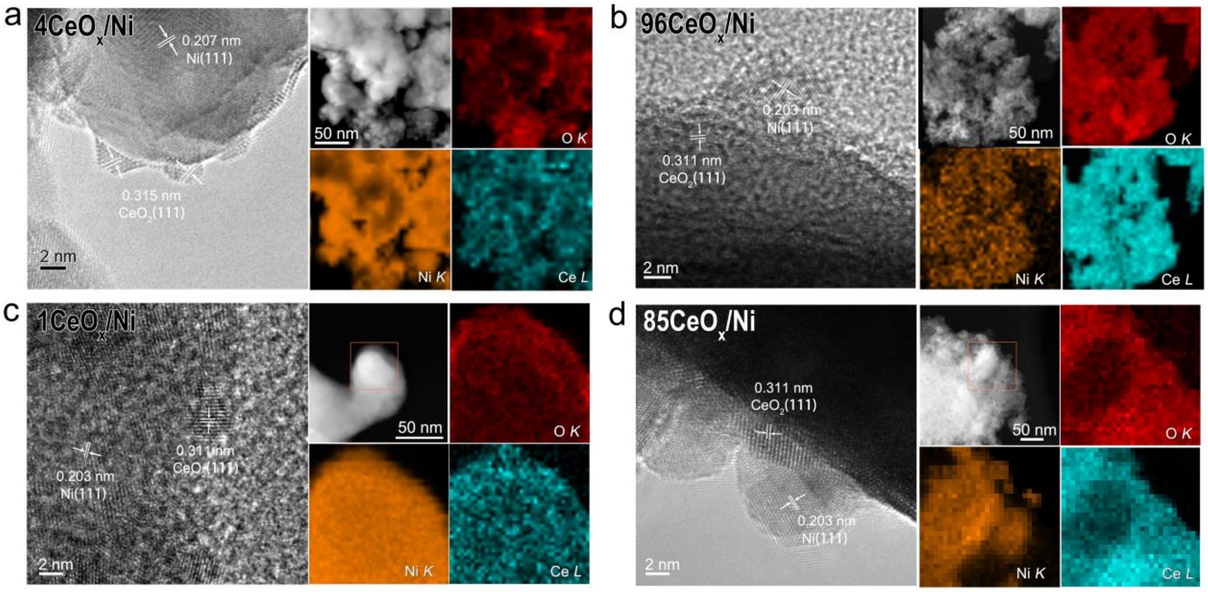


**Supplementary Fig. 2. TEM characterization images of n$CeO_x$/Ni catalysts.** HRTEM images, HAADF-STEM images, and the corresponding EDS mapping of **a** 4$CeO_x$/Ni, **b** 96$CeO_x$/Ni, **c** 1$CeO_x$/Ni, and **d** 85$CeO_x$/Ni catalysts.

**Supplementary Note 1:** The HRTEM images show that the $CeO_x$ cluster, approximately 2~3 nm in size, are supported on Ni over the inverse 4$CeO_x$/Ni and 1$CeO_x$/Ni catalysts. The particle size of Ni on the conventional 96$CeO_x$/Ni catalyst is 6 nm, increasing to approximately 8 nm for the 85$CeO_x$/Ni catalyst. The EDS mapping reveals that Ce is well-dispersed on Ni over the inverse 4$CeO_x$/Ni and 1$CeO_x$/Ni catalysts, while Ni is uniformly dispersed on $CeO_2$ over the 96$CeO_x$/Ni and 85$CeO_x$/Ni catalysts.

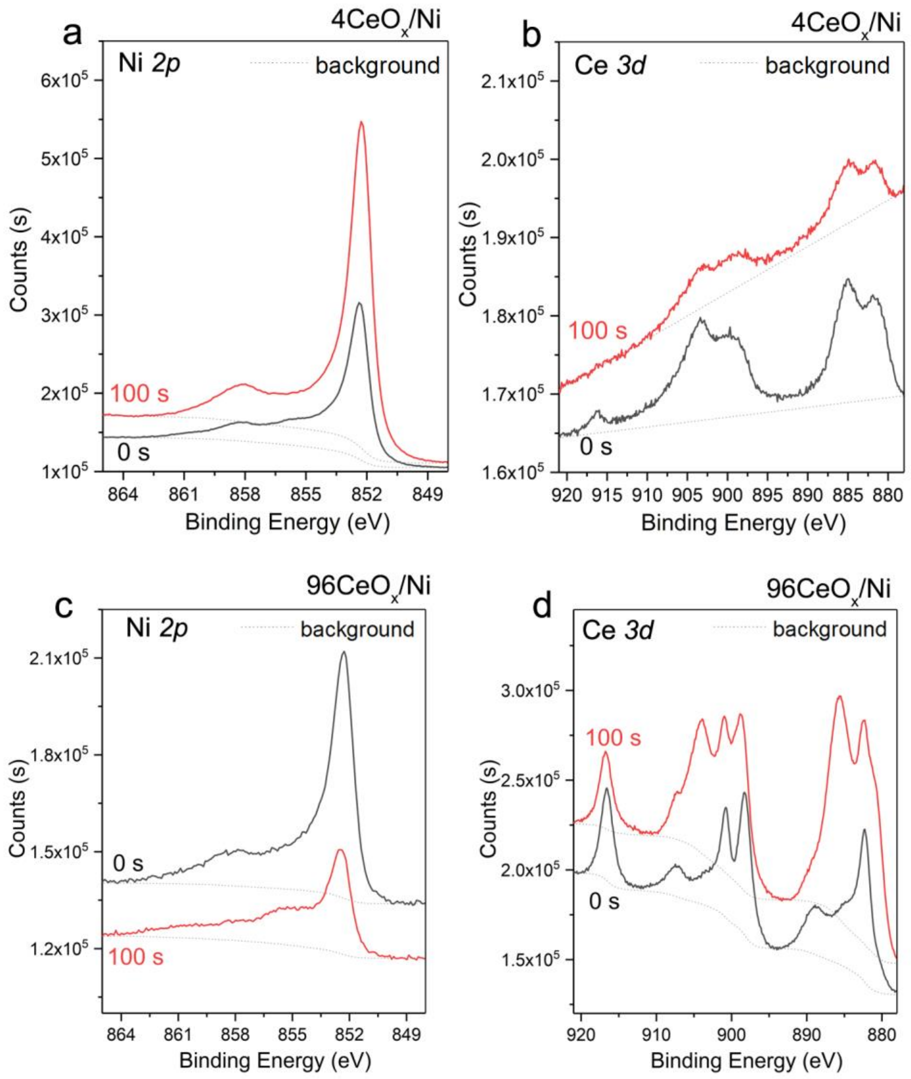


**Supplementary Fig. 3. Surface analysis study of $4CeO_x$/Ni and $96CeO_x$/Ni catalysts. a,c** Ni *2p* and **b,d** Ce *3d* XPS spectra of the $4CeO_x$/Ni and $96CeO_x$/Ni catalysts before and after 100 seconds of $Ar^+$ ions sputtering, respectively.

**Supplementary Note 2:** In situ XPS analysis combined with $Ar^+$ ion sputtering treatment can give element distribution information along the depth direction with the exposure of the subsurface layer of the catalyst. For the $4CeO_x$/Ni catalyst, the intensity of Ni peak at 852.3 eV (refer to baseline, the same below) increases from $2.0\times10^5$ to $4.2\times10^5$ counts after sputtering for 100 seconds (Supplementary Fig. 3a). Simultaneously, the corresponding Ce element signal decrease from $1.6\times10^4$ to $0.7\times10^4$ counts (calculated based on the characteristic peak at 885.1 eV, Supplementary Fig. 3b). The trend indicates that the relative amount of Ni elements increases from surface to sub-surface of the inverse $4CeO_x$/Ni catalyst after $Ar^+$ ion sputtering treatment, while the amount of Ce elements corresponding decreases. In contrast, the Ni signal (peak at 852.3 eV) decreases from $0.7\times10^5$ to $0.3\times10^5$ counts (Supplementary Fig. 3c) over the $96CeO_x$/Ni catalysts, and the corresponding Ce signal (peak at 882.3 eV) increases from $0.8\times10^5$ to $1.2\times10^5$ counts (Supplementary Fig. 3d), which show that the relative amount of Ce elements increases from surface to sub-surface of the conventional $96CeO_x$/Ni catalyst after $Ar^+$ ion sputtering treatment of the conventional $96CeO_x$/Ni catalyst, while the amount of Ni elements corresponding decreases. It is worth noting that the significant chemical valence changes of the Ce species in the $4CeO_x$/Ni and $96CeO_x$/Ni catalysts after $Ar^+$ ion sputtering treatment can be attributed to removal of oxygen elements by $Ar^+$ ion beam sputtering, which lead to the reduction of $CeO_2$.[1]

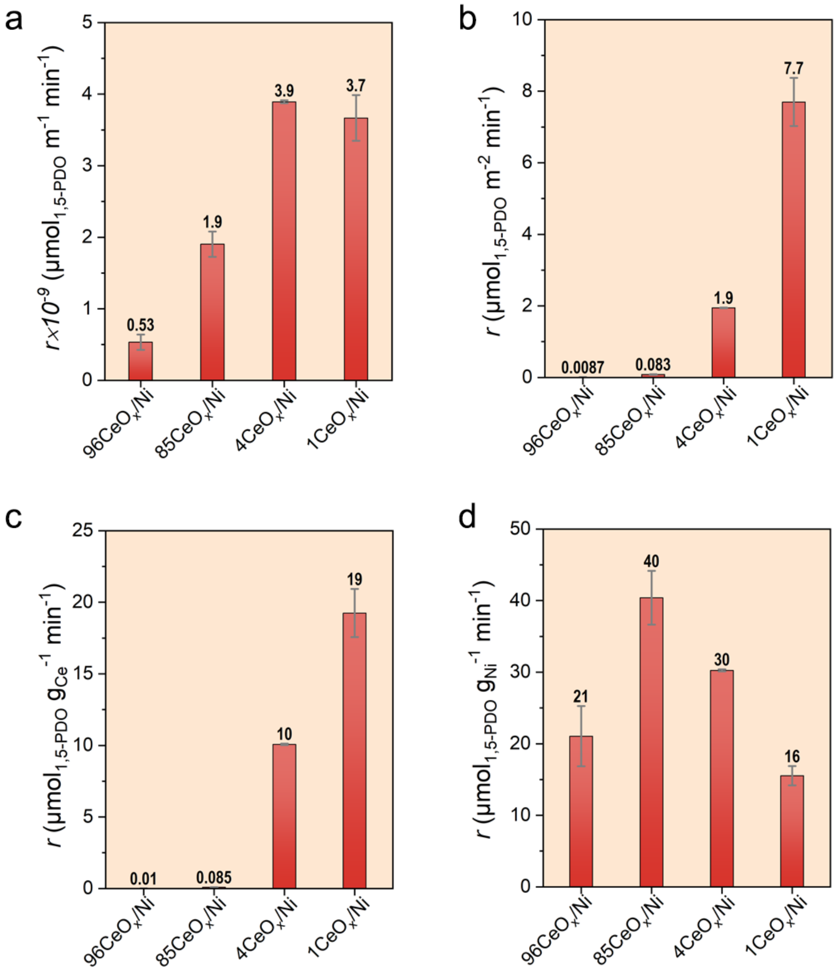


**Supplementary Fig. 4. The normalized reaction rates.** The reaction rates of THFA hydrogenolysis over $1CeO_x$/Ni, $4CeO_x$/Ni, $85CeO_x$/Ni, and $96CeO_x$/Ni catalysts are normalized by their **a** interfacial perimeter length, **b** surface areas, **c** Ce mass, and **d** Ni mass.

**Supplementary Note 3:** As shown in Supplementary Fig. 4a, the reaction rate is normalized by their interfacial perimeter length (Supplementary Table 2), which can be estimated from the size of $CeO_x$ or Ni cluster determined by HRTEM analysis.[2]

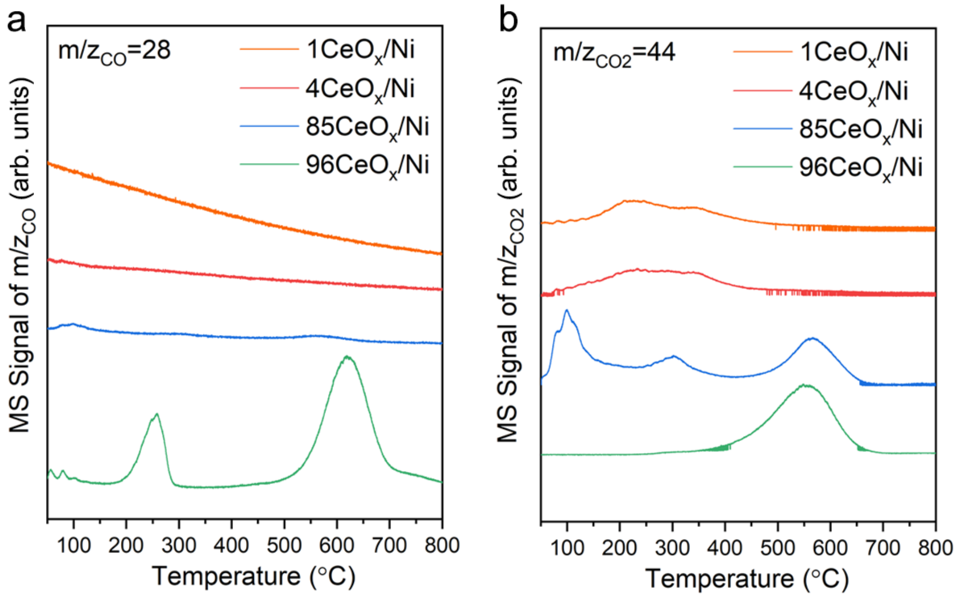


**Supplementary Fig. 5**. **CO-TPD-MS profiles of 1CeO$_x$/Ni, 4CeO$_x$/Ni, 85CeO$_x$/Ni, and 96CeO$_x$/Ni catalysts. a** CO signal of m/z=28; **b** $CO_2$ signal of m/z=44.

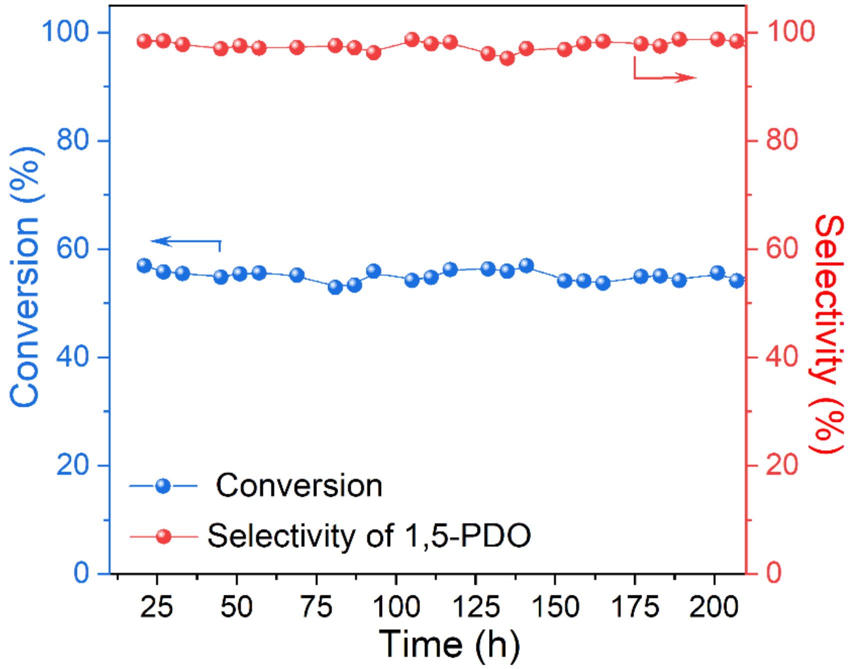


**Supplementary Fig. 6**. **Catalyst stability experiment in a fixed-bed reactor over a 4$CeO_x$/Ni catalyst.** Before the reaction, the calcined catalyst is pre-reduced in a $H_2$/$N_2$ flow. Reaction conditions: calcined catalyst: 13 g; THFA (solvent-free): 0.04 mL min$^{-1}$; 160 °C; 4.0 MPa $H_2$; gas-flow rate: 50 mL min$^{-1}$.

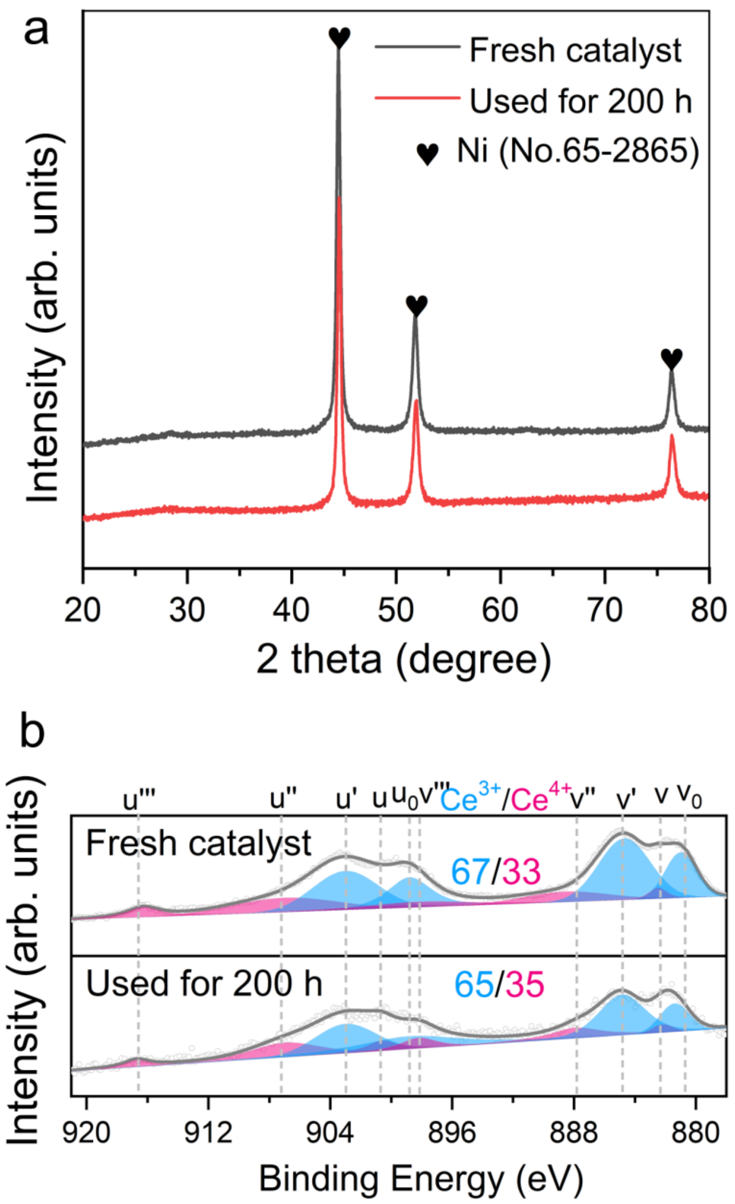


**Supplementary Fig. 7. Structural analysis of the spent 4CeO$_x$/Ni catalyst. a** XRD patterns and **b** XPS spectra of Ce *3d* spectra of the 4CeO$_x$/Ni catalyst before and after a 200-hour reaction in a fixed-bed reactor.

**Supplementary Note 4:** The average grain sizes of metallic Ni in 4CeO$_x$/Ni catalysts before and after the reaction are calculated to be approximately 23 nm and 22 nm (Supplementary Fig. 7a), respectively, demonstrating the stability in the crystal size of metallic Ni due to the surface CeO$_x$ cluster inhibiting the migration and agglomeration of Ni species. The relative concentration of $Ce^{3+}$ species is calculated to be 67% and 65% before and after reaction (Supplementary Fig. 7b), indicating the high stability of $Ce^{3+}$ species on the inverse 4CeO$_x$/Ni catalyst during the reaction.

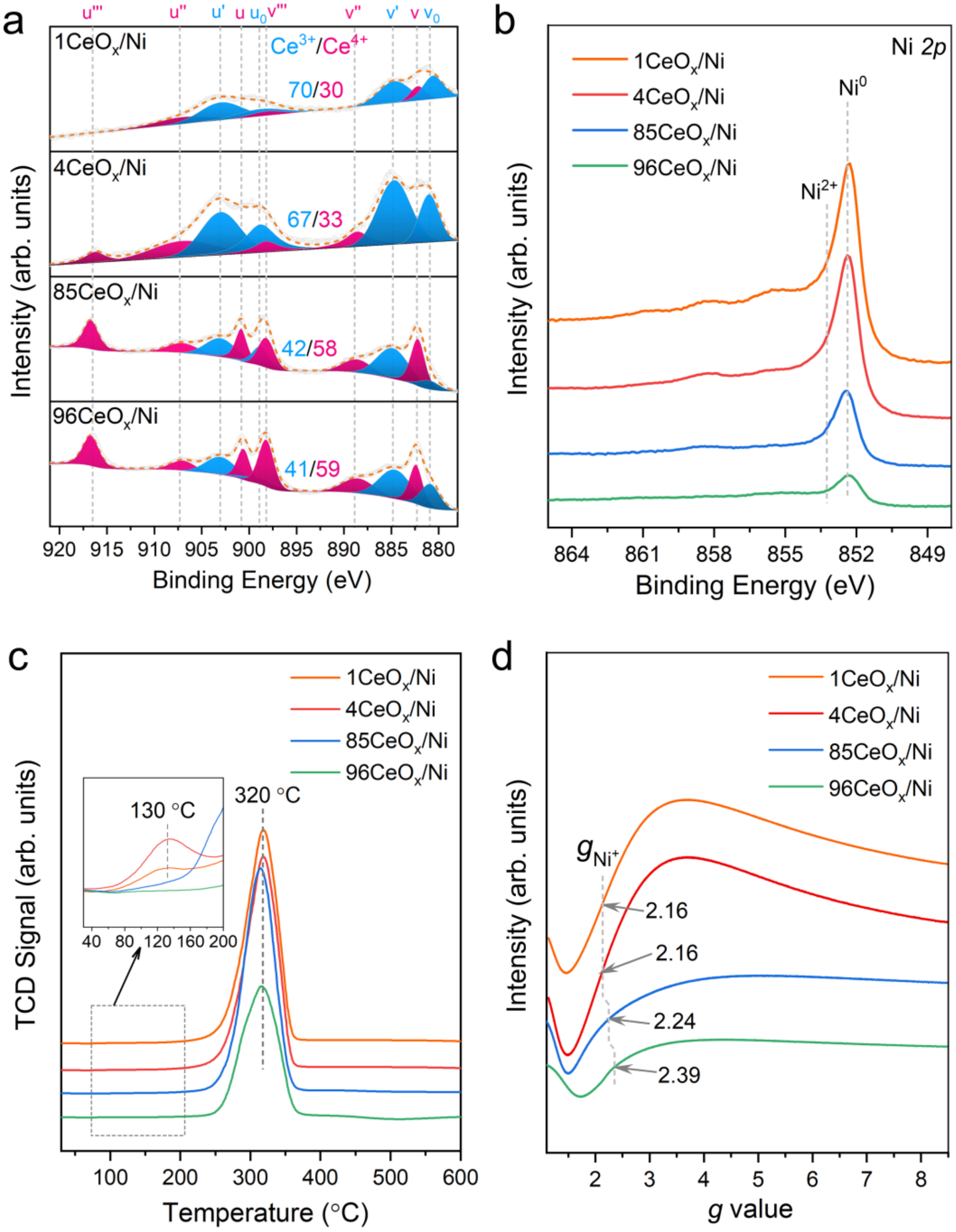


**Supplementary Fig. 8. Structural analysis of $1CeO_x/Ni$, $4CeO_x/Ni$, $85CeO_x/Ni$, and $96CeO_x/Ni$ catalysts.** Quasi in situ XPS spectra of **a** Ce *3d* and **b** Ni *2p* spectra of the $nCeO_x/Ni$ catalysts. **c** $H_2$-TPR profiles and **d** EPR spectra of the $nCeO_x/Ni$ catalysts.

**Supplementary Note 5:** As shown in Supplementary Fig. 8a, the Ce *3d* XPS spectra can be decomposed into eight peaks representing the $Ce^{4+}$ species (v, v″, v‴, u, u″, and u‴) and $Ce^{3+}$ species ($v^0$, v′, $u^0$, and u′), and their relative concentration of Ce species are calculated by integrating the corresponding peak areas.

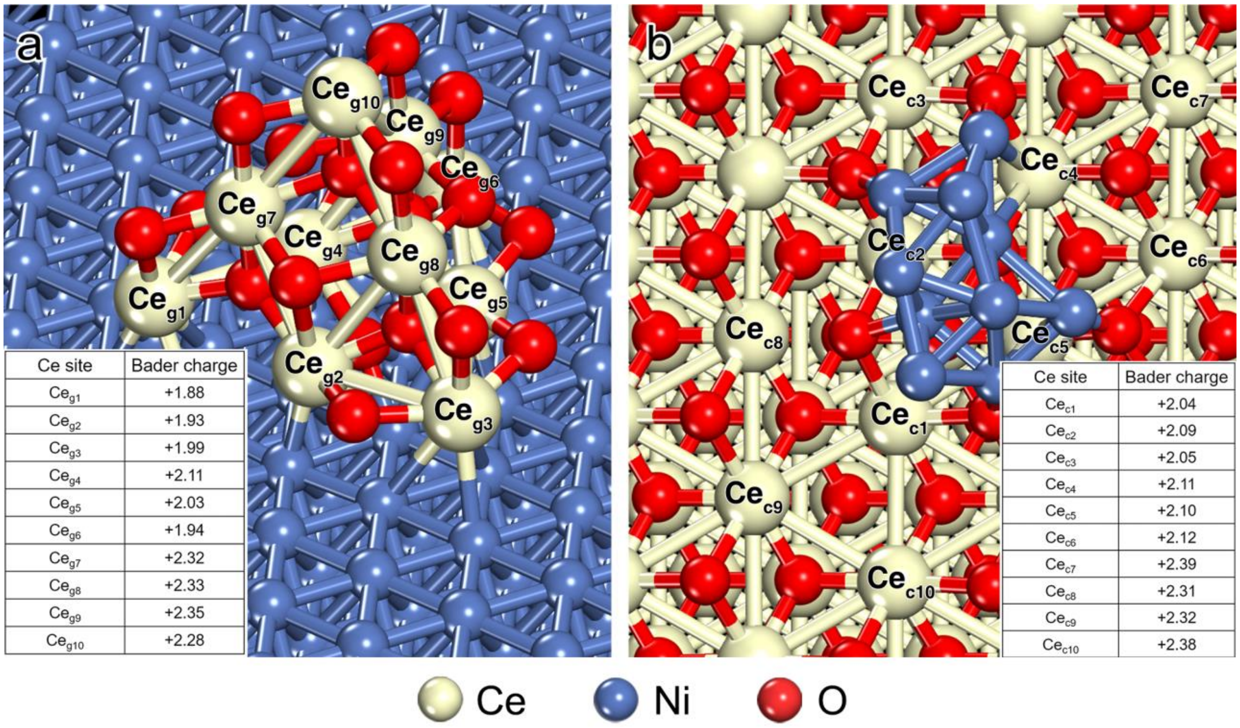


**Supplementary Fig. 9. Bader charge analysis of Ce atoms. a** In $Ce_{10}O_{19}$/Ni(111), bulk Ce is labeled as $Ce_{g7}$–$Ce_{g10}$, interfacial Ce without adjacent oxygen vacancy is labeled as $Ce_{g3}$–$Ce_{g6}$, and interfacial Ce with an adjacent oxygen vacancy is labeled as $Ce_{g1}$–$Ce_{g2}$, respectively. **b** In $Ni_{10}$/$CeO_2$(111), bulk Ce is labeled as $Ce_{c7}$–$Ce_{c10}$, interfacial Ce without adjacent oxygen vacancy is labeled as $Ce_{c2}$–$Ce_{c6}$, and interfacial Ce with an adjacent oxygen vacancy is labeled as $Ce_{c1}$, respectively. Bader charge of Ce atoms in $Ce_{10}O_{19}$/Ni(111) and $Ni_{10}$/$CeO_2$(111) supercells is relation to the charge of a neutral Ce atom (12.00 a.u.).

**Supplementary Note 6:** The Bader charges of $Ce_{bulk}$, $Ce(–O)_{interface}$, and $Ce(–V_O)_{interface}$ in $Ce_{10}O_{19}$/Ni(111) are calculated to be +2.32, +2.02, and +1.90 based on the average charge of the corresponding Ce atoms. Similarly, the Bader charges of $Ce_{bulk}$, $Ce(–O)_{interface}$, and $Ce(–V_O)_{interface}$ in $Ni_{10}$/$CeO_2$(111) are calculated to be +2.35, +2.09, and +2.04, respectively.

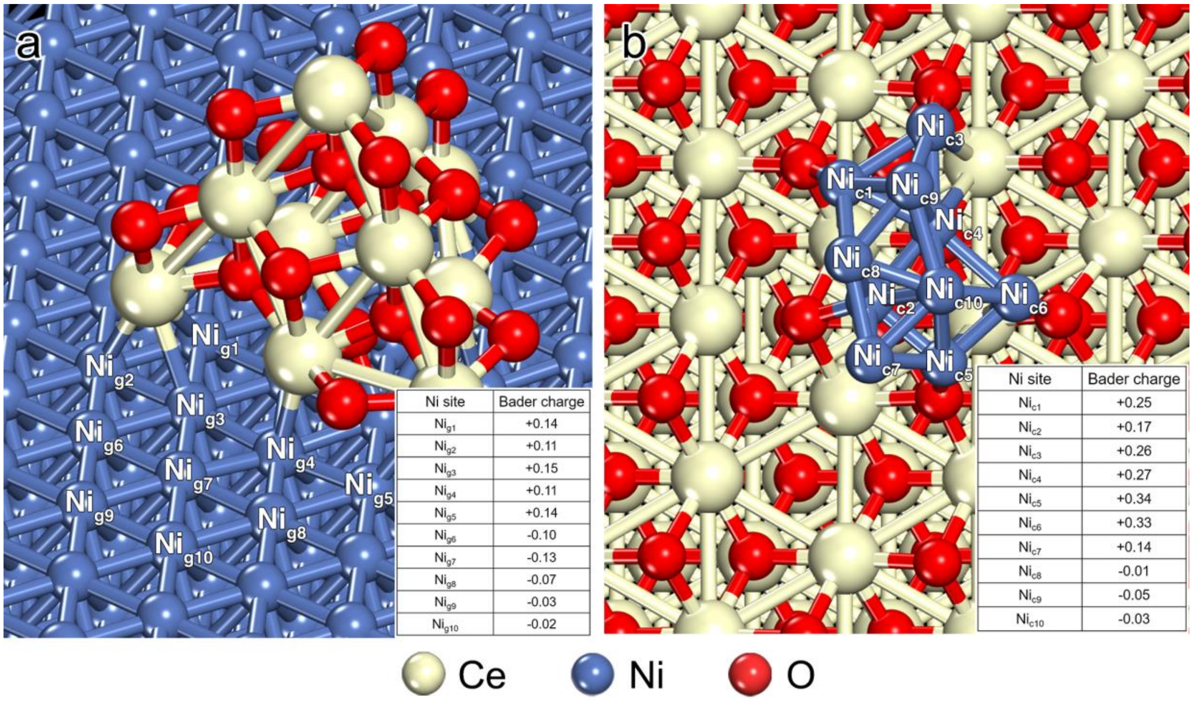


**Supplementary Fig. 10**. **Bader charge analysis of Ni atoms. a** In $Ce_{10}O_{19}$/Ni(111), interfacial Ni and bulk Ni are labelled as $Ni_{g1}$–$Ni_{g5}$ and $Ni_{g6}$–$Ni_{g10}$, respectively. **b** In $Ni_{10}$/$CeO_2$(111), interfacial Ni and bulk Ni are labelled as $Ni_{c1}$–$Ni_{c6}$ and $Ni_{c7}$–$Ni_{c10}$, respectively. Bader charge of Ni atoms in $Ce_{10}O_{19}$/Ni(111) and $Ni_{10}$/$CeO_2$(111) supercells is relation to the charge of a neutral Ni atom (10.00 a.u.).

**Supplementary Note 7:** The Bader charges of $Ni_{bulk}$ and $Ni(\text{-}Ce)_{interface}$ over $Ce_{10}O_{19}$/Ni(111) are calculated to be –0.07 and +0.13 based on the average charge of the corresponding Ni atoms. For $Ni_{10}$/$CeO_2$(111), the Bader charges are calculated to be –0.03 for $Ni_{bulk}$ and +0.25 for $Ni(\text{-}Ce)_{interface}$.

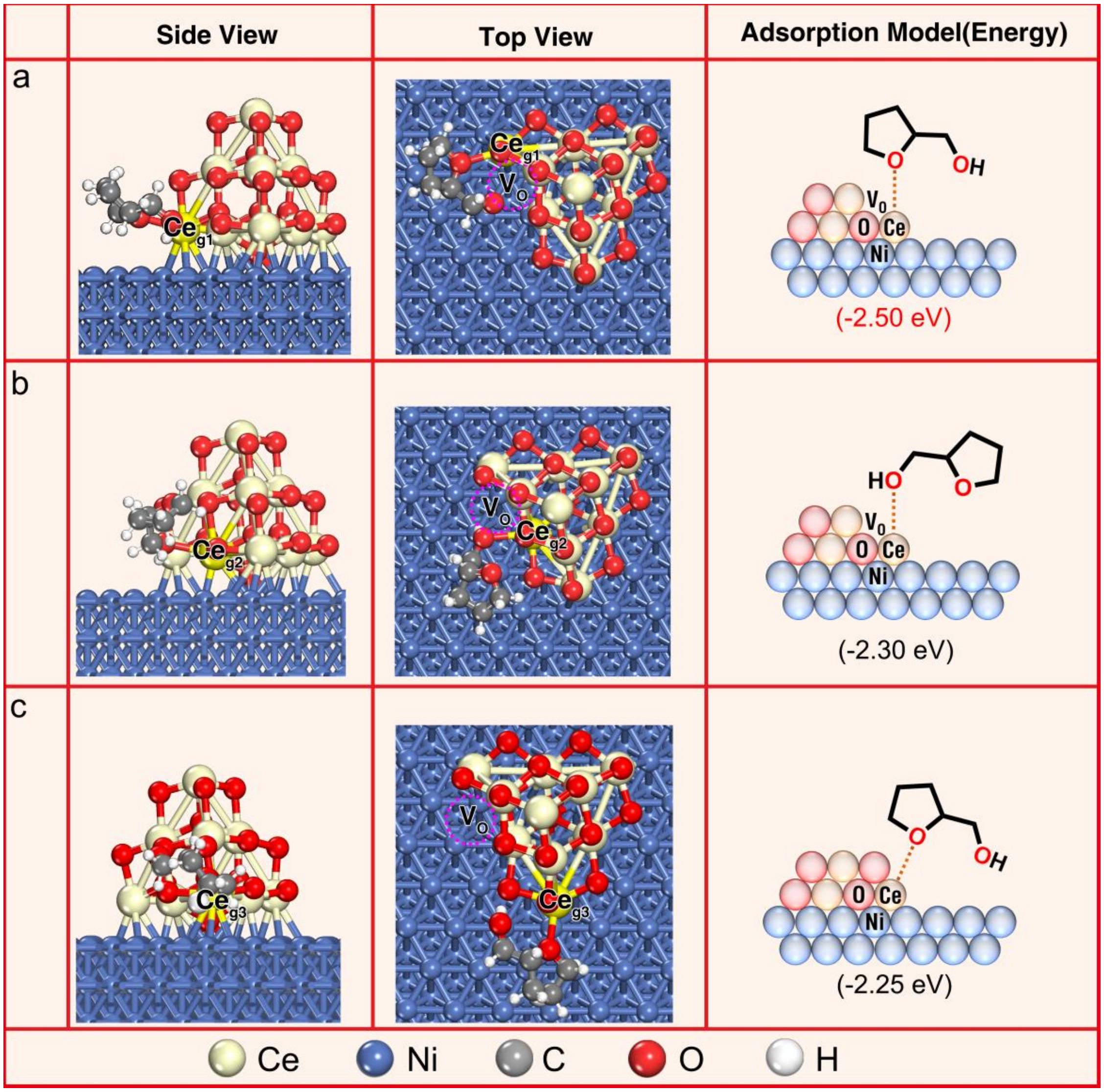


**Supplementary Fig. 11**. **The calculated structures of absorbed THFA on inverse $Ce_{10}O_{19}$/Ni(111). a** Etheric oxygen atom is adsorbed at the interfacial Ce sites ($Ce_{g1}$) adjacent to oxygen vacancy. **b** Hydroxyl oxygen is adsorbed at the interfacial Ce sites ($Ce_{g2}$) adjacent to oxygen vacancy. **c** Etheric oxygen atom is adsorbed at the Ce sites ($Ce_{g3}$).

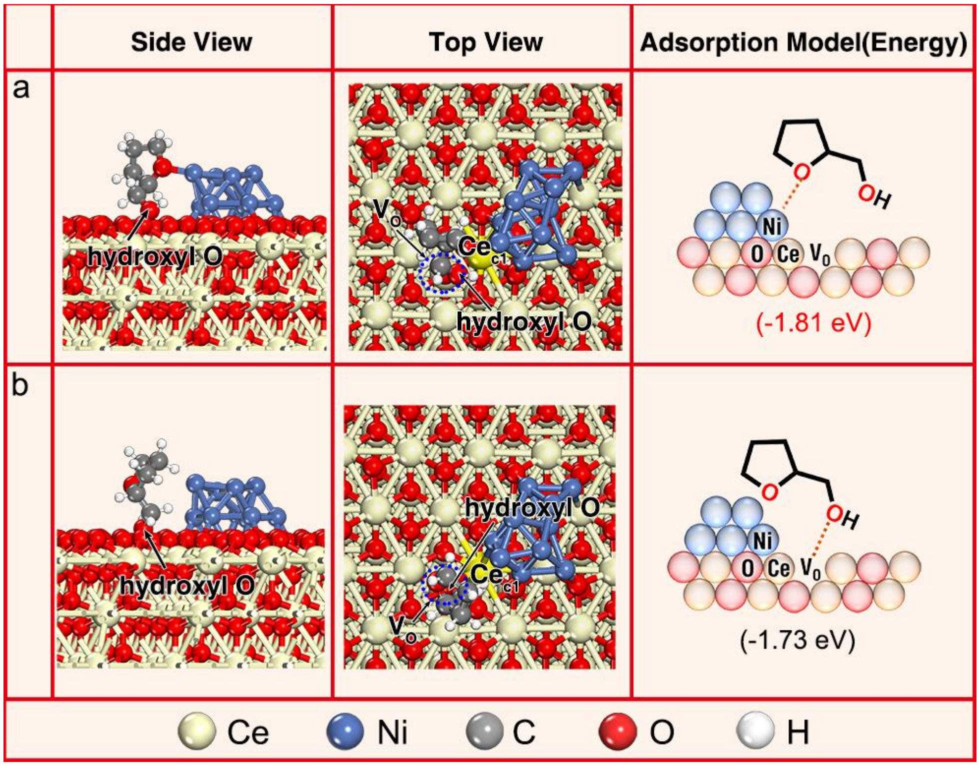


**Supplementary Fig. 12**. **The calculated structures of absorbed THFA on conventional $Ni_{10}/CeO_2(111)$. a** Etheric oxygen is adsorbed at the interfacial Ni sites, and **b** hydroxyl oxygen is adsorbed at the interfacial oxygen vacancy.

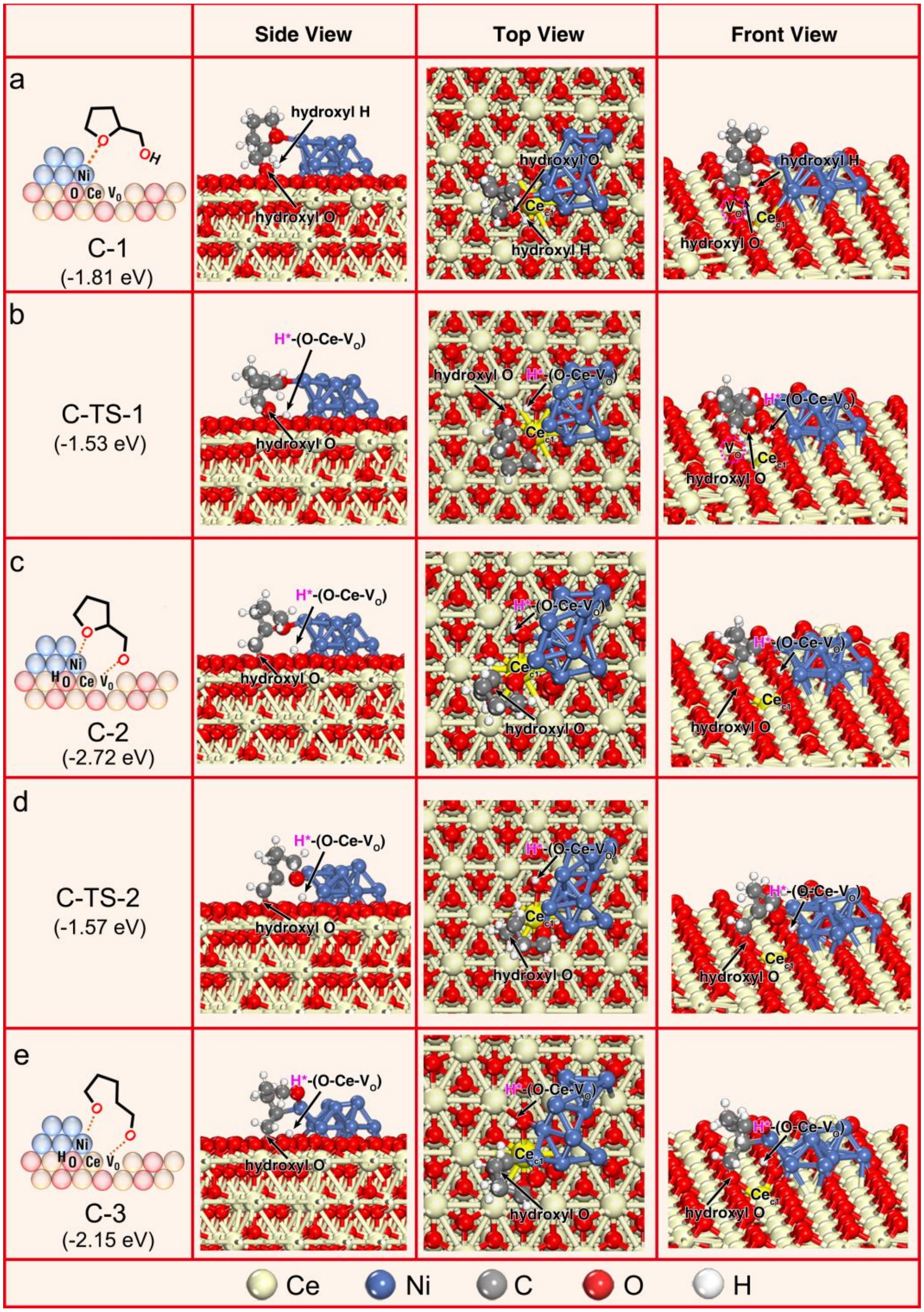


**Supplementary Fig. 13**. **DFT calculations for the cleavage of C2–O bond in THFA on $Ni_{10}/CeO_2(111)$ when etheric oxygen is adsorbed at the interfacial Ni sites.** **a** Etheric oxygen is adsorbed at the interfacial Ni sites. **b** The hydroxyl O–H bond is cleaved, and the dissociated H is adsorbed on oxygen adjacent to oxygen vacancy. **c** Etheric oxygen and hydroxyl oxygen are adsorbed at the interfacial Ni sites and oxygen vacancy, respectively. **d** The etheric C2–O bond is cleaved. **e** Oxygen atoms are adsorbed at the interfacial Ni sites and oxygen vacancy, respectively.

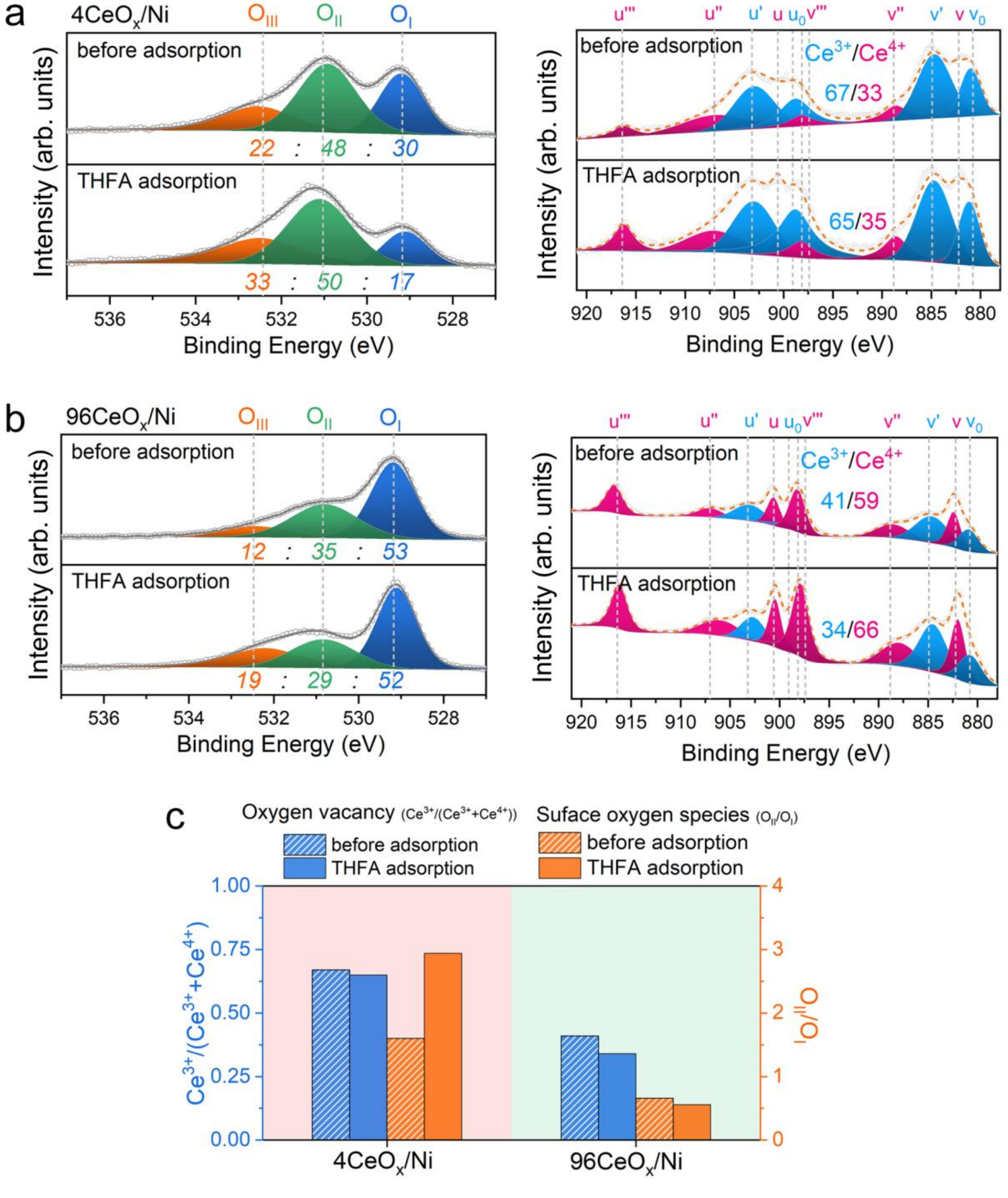


**Supplementary Fig. 14. Adsorption experiments of THFA on 4CeO$_x$/Ni and 96CeO$_x$/Ni catalysts.** Quasi in situ XPS spectra of O *1s* and Ce *3d* of **a** 4CeO$_x$/Ni and **b** 96CeO$_x$/Ni catalysts before and after THFA adsorption. **c** The proportions of the corresponding oxygen species on the 4CeO$_x$/Ni and 96CeO$_x$/Ni catalysts ($O_I$ and $O_{II}$ represent lattice oxygen and oxygen species are associated with oxygen vacancy).

**Supplementary Note 8:** To study the adsorption sites for the oxygenic group of THFA, the oxygen species on the catalyst were analyzed by quasi in situ XPS. The reduced catalyst was first impregnated in THFA and then vacuumed overnight to remove the physical adsorbed THFA for the following XPS analysis. As shown in Supplementary Fig. 14a,b, the XPS spectra of O *1s* can be deconvoluted into three peaks at 529.1 eV, 530.9 eV, and 532.4 eV, which are assigned to lattice oxygen of $CeO_2$ ($O_I$), the surface-adsorbed oxygen species associated with oxygen vacancy ($O_{II}$), and adsorbed water ($O_{III}$), respectively.[2,3] The relative content of $O_{II}$ is calculated by its corresponding area ratio to $O_I$, since the lattice oxygen species ($O_I$) is stable before and after THFA adsorption. As shown in Supplementary Fig. 14c, after THFA adsorption on the inverse 4CeO$_x$/Ni catalyst, the $O_{II}/O_I$ ratio increases from 1.6 to 2.9 with no remarkable charge of oxygen vacancy (calculated by the ratio of $Ce^{3+}/(Ce^{3+}+Ce^{4+})$). It can be deduced that the etheric oxygen of THFA preferred to adsorb at the Ce(−V$_O$)$_{interface}$ sites adjacent to oxygen vacancy on the inverse catalyst. In contrast, the adsorption of THFA on 96CeO$_x$/Ni catalyst gave rise to the decrease of the $O_{II}/O_I$ ratio from 0.6 to 0.5 and the diminishment of oxygen vacancy from 41% to 34%, which demonstrate that the deprotonated hydroxyl atom of THFA is strongly adsorbed at the oxygen vacancy.

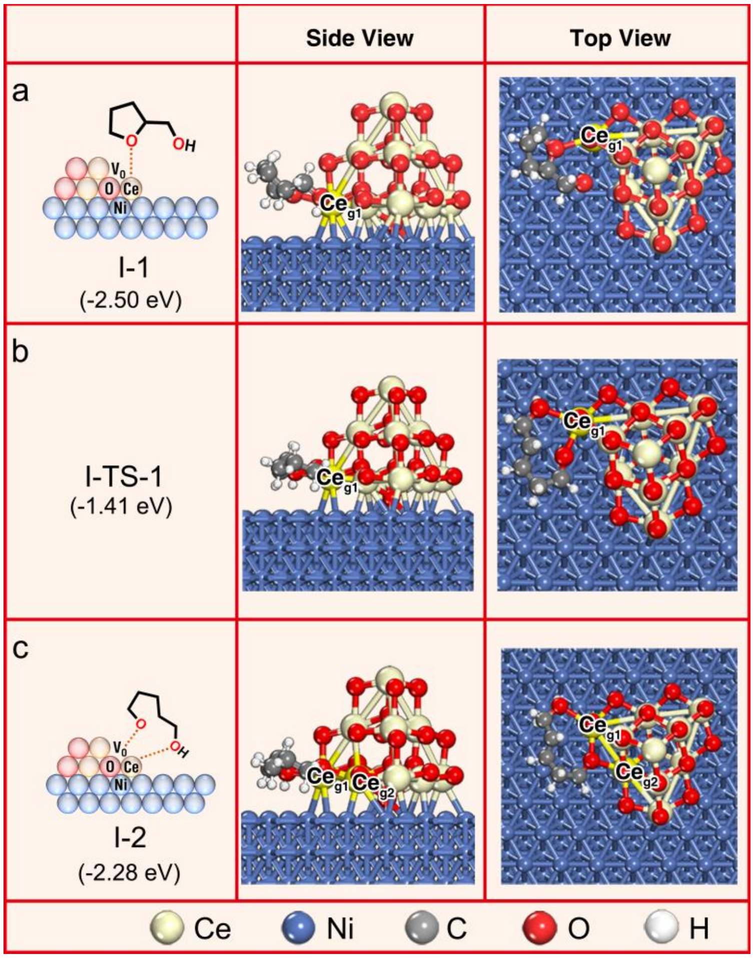


**Supplementary Fig. 15. DFT calculations for the cleavage of C2–O bond in THFA on $Ce_{10}O_{19}$/Ni(111) when etheric oxygen atom is adsorbed at the interfacial Ce sites adjacent to oxygen vacancy. a** Etheric oxygen is adsorbed at the interfacial Ce sites adjacent to oxygen vacancy. **b** Etheric C2–O bond is cleaved. **c** Etheric oxygen and hydroxyl oxygen are adsorbed at the oxygen vacancy and interfacial Ce sites adjacent to oxygen vacancy, respectively.

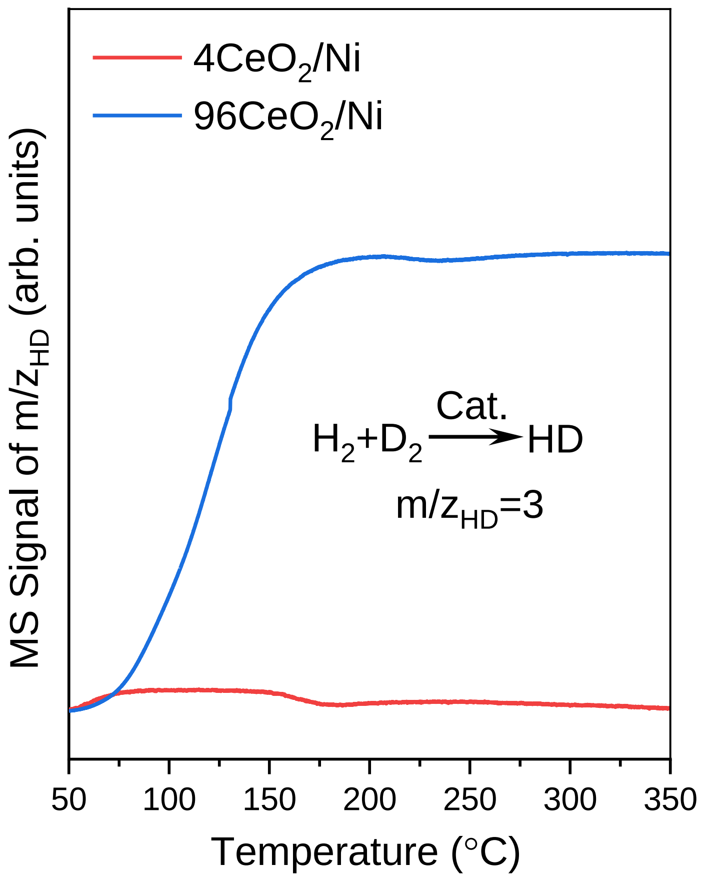


**Supplementary Fig. 16. H–D exchange experiments.** HD profiles of gas-phase isotopic H–D exchange over $4CeO_x$/Ni and $96CeO_x$/Ni catalysts.

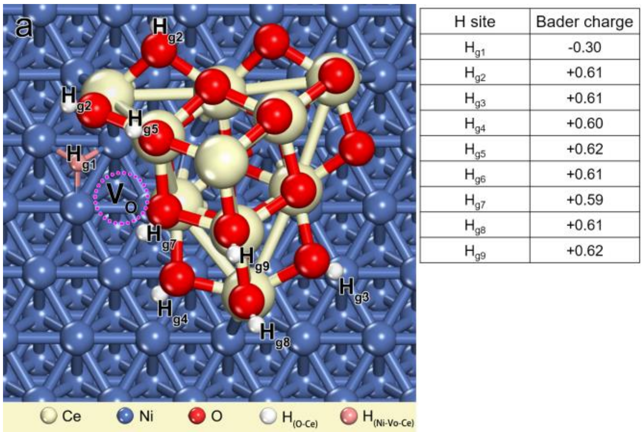


| H site | Bader charge |
|---|---|
| $H_{g1}$ | -0.30 |
| $H_{g2}$ | +0.61 |
| $H_{g3}$ | +0.61 |
| $H_{g4}$ | +0.60 |
| $H_{g5}$ | +0.62 |
| $H_{g6}$ | +0.61 |
| $H_{g7}$ | +0.59 |
| $H_{g8}$ | +0.61 |
| $H_{g9}$ | +0.62 |

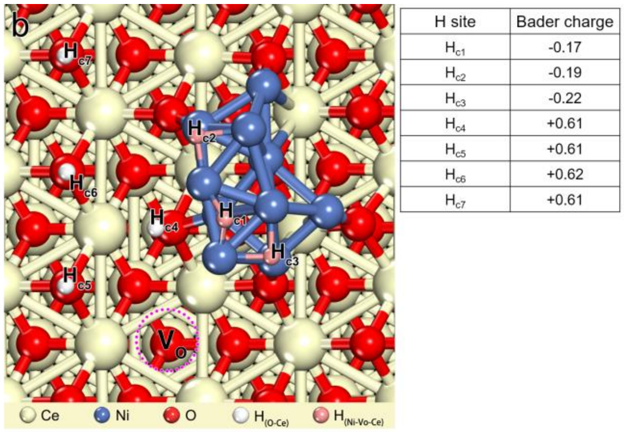


| H site | Bader charge |
|---|---|
| $H_{c1}$ | -0.17 |
| $H_{c2}$ | -0.19 |
| $H_{c3}$ | -0.22 |
| $H_{c4}$ | +0.61 |
| $H_{c5}$ | +0.61 |
| $H_{c6}$ | +0.62 |
| $H_{c7}$ | +0.61 |

**Supplementary Fig. 17. Bader charge analysis of H species.** Bader charge of H atoms in **a** $Ce_{10}O_{19}$/Ni(111) and **b** $Ni_{10}$/$CeO_2$(111) supercells is relation to the charge of a neutral H atom (1.00 a.u.). The H atoms bonded with Ni atom adjacent to oxygen vacancy (labeled as $H_{g1}$ and $H_{c1}$–$H_{c3}$) and with O atom of $CeO_x$ (labeled as $H_{g2}$–$H_{g9}$ and $H_{c4}$–$Ni_{c7}$) are denoted as H*–Ni(–Ce)$_{interface}$ and H*–(O–Ce)$_{interface}$, respectively.

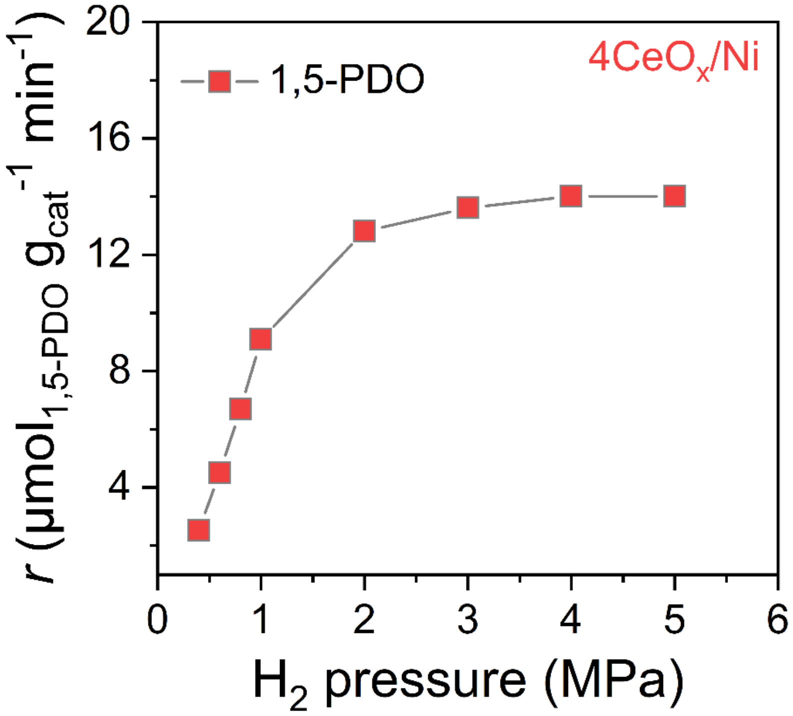


**Supplementary Fig. 18. The kinetic analysis.** The kinetic effects of $H_2$ pressure on the catalytic hydrogenolysis of THFA to 1,5–PDO over inverse $4CeO_x/Ni$ catalyst. The experiments are conducted in a batch reactor. Reaction conditions: THFA, 0.5 g; catalyst, 0.25 g; ethanol, 5 mL; 160 °C; 10 h.

**Supplementary Table 1**. Catalytic performance of different catalysts for the conversion of THFA to 1,5–PDO.

| Entry | Catalyst | Feed conditions | Stability (conversion loss) | Conversion (%) | Selectivity (%) | Yield (%) | Ref. |
|---|---|---|---|---|---|---|---|
| 1 | 4wt.% Rh-$ReO_x$/C (Re/Rh=0.5) | 5 wt. % THFA aqueous solution; 4 MPa $H_2$; catalyst/reactant = 1/9 (w./w.); 120 °C | fixed-bed reactor, 120 h (42%→32%) | 47 | 97 | 46 | 4 |
| 2 | 4wt.%Rh-$ReO_x$/$SiO_2$ (Re/Rh=0.13) | 20 mL aqueous solution of 60 wt%; 8 MPa $H_2$; 0.2 g catalyst; 120 °C; 12 h | batch reactor, 5 cycles (79%→65%) | 79 | 94 | 74 | 5 |
| 3 | 4wt.% Ir-$VO_x$/$SiO_2$ (V/Ir=0.11) | 20 wt. % THFA aqueous solution 0.04 mL/min; 6 MPa $H_2$; 2 g catalyst; 80 °C | fixed-bed reactor, 30 h (58%→20%) | 58 | 89 | 52 | 6 |
| 4 | 4wt.% Ir-$ReO_x$/$SiO_2$ (Re/Ir=3) | 1 g THFA; 4 mL water; 8 MPa $H_2$; 0.15 g catalyst; 100 °C; 2 h | - | 60 | 94 | 56 | 7 |
| 5 | 3wt.% Pt-5wt.% $WO_x$/$ZrO_2$ | 1.5 g THFA; 5 mL 2-propanol; 5 MPa $H_2$; 0.05 g catalyst; 150 °C; 15 h | - | 55 | 64 | 35 | 8 |
| 6 | 1wt.% Ru-Ni/$Y_2O_3$ (Ni/Y=2.5) | 1 g THFA; 3 mL 2-propanol; 2 MPa $H_2$; 0.05 g catalyst; 150 °C; 40 h | - | 93 | 87 | 81 | 9 |
| 7 | 20wt.% Ni/$Al_2O_3$ | 5 wt. % THFA aqueous solution; 250 °C | - | 17 | 59 | 10 | 10 |
| 8 | 10wt.% Ni/ZSM-5 | 5 g THFA; 95 mL water; 4 MPa $H_2$; 0.5 g catalyst; 250 °C; 4 h | batch reactor, 1 cycle (93%→74%) | 93 | 4 | 4 | 11 |
| 9 | 10wt.% Ni-$WO_x$/$SiO_2$ (W/Ni=1) | 3 g THFA; 57 mL water; 3.4 MPa $H_2$; 0.3 g catalyst; 250 °C; 4 h | batch reactor, 2 cycles (39%→29%) | 39 | 40 | 16 | 12 |
| 10 | 40wt.% Ni-La (Ni/La=2.5) | 1.3 wt.% THFA solution (isopropanol); 3 MPa $N_2$; 3 g catalyst; 140 °C | fixed-bed reactor, 192 h (84%→80%) | 84 | 88 | 73 | 13 |
| 11 | $NiPr_{1.2}$/$Al_2O_3$ | 0.18 g THFA; 10 mL 2-propanol; 6 MPa $H_2$, 0.1 g catalyst; 12 h | batch reactor, 2 cycles (50%→43%) | 50 | 89 | 45 | 14 |
| 12 | 4$CeO_x$/Ni | 100 wt. % THFA (solvent free) 0.02 mL/min; 4 MPa $H_2$; 13 g calcined catalyst; 160 °C | fixed-bed reactor, 200 h (95%→95%) | 95 | 98 | 93 | This work |

**Supplementary Table 2**. Physical and chemical characterizations of n$CeO_x$/Ni catalysts.

| Sample | Ni content[a] (wt %) | Ce content[a] (wt %) | $S_{BET}$[b] ($m^2$ $g^{-1}$) | $V_{Pore}$[b] ($m^3$ $g^{-1}$) | $D_{Pore}$[b] (nm) | Interfacial perimeter length[c] (m $g^{-1}$) |
|---|---|---|---|---|---|---|
| 96$CeO_x$/Ni | 3.8 | 78.3 | 92 | 0.11 | 4 | $1.5\times10^9$ |
| 85$CeO_x$/Ni | 14.6 | 69.5 | 71 | 0.09 | 5 | $3.1\times10^9$ |
| 4$CeO_x$/Ni | 96.5 | 2.9 | 15 | 0.11 | 30 | $7.5\times10^9$ |
| 1$CeO_x$/Ni | 99.1 | 0.8 | 2 | 0.03 | 73 | $4.2\times10^9$ |

[a]Actual Ni and Ce contents were determined by ICP-MS. [b]Determined by $N_2$ physisorption. [c]The total length of the nickel-ceria interfacial perimeter is calculated from the size of $CeO_x$ clusters on inverse 1$CeO_x$/Ni and 4$CeO_x$/Ni catalysts, and Ni clusters on conventional catalyst, respectively[15].

The perimeter length was calculated using the following equation:

$$Perimeter\ length = \frac{m \times \pi \times d}{\rho \times \frac{1}{2} \times \frac{4}{3} \times \pi \times (\frac{d}{2})^3}$$

Here, the model of hemispherical $CeO_x$ or Ni cluster supported on Ni or $CeO_2$ is used. *d* represents the particle size of $CeO_x$ or Ni cluster determined from the HRTEM analysis. *m* and *ρ* are the loading content and the density of $CeO_2$ or Ni, respectively.

**Supplementary Note 9:** 4$CeO_x$/Ni catalyst has a reaction rate normalized by mass of catalyst (29.2 $\mu mol_{1,5\text{-}PDO}$ $g^{-1}$ $min^{-1}$), which is approximately twice as high as that of the 1$CeO_x$/Ni catalyst (15.4 $\mu mol_{1,5\text{-}PDO}$ $g^{-1}$ $min^{-1}$). This higher rate can be attributed to the longer interfacial perimeter length of the 4$CeO_x$/Ni catalyst, which is $7.5\times10^9$ m $g^{-1}$, about twice that of the 1$CeO_x$/Ni catalyst at $4.2\times10^9$ m $g^{-1}$.

**Supplementary Table 3**. Selective hydrogenolysis of THFA over various catalysts in a fixed-bed reactor[a]

| Catalyst | Conversion (%) | Selectivity (%) | | | |
|---|---|---|---|---|---|
| | | 1,5–PDO | 1–butanol | 1–pentanol | MTHF |
| 96CeO$_x$/Ni | 2 | 98 | 1 | <1 | <1 |
| | 3 | 96 | 2 | <1 | <1 |
| | 2 | 94 | 2 | <1 | <1 |
| 85CeO$_x$/Ni | 14 | 97 | 1 | <1 | <1 |
| | 17 | 97 | 1 | <1 | <1 |
| | 15 | 99 | 1 | <1 | <1 |
| 4CeO$_x$/Ni | 63 | 98 | 2 | <1 | <1 |
| | 63 | 99 | 1 | <1 | <1 |
| | 64 | 97 | 2 | <1 | <1 |
| 1CeO$_x$/Ni | 35 | 97 | 1 | <1 | <1 |
| | 32 | 92 | 1 | <1 | <1 |
| | 39 | 98 | 1 | <1 | <1 |
| $CeO_2$ | <1 | - | - | - | - |
| | <1 | - | - | - | - |
| | <1 | - | - | - | - |
| Ni | <1 | - | - | - | - |
| | <1 | - | - | - | - |
| | <1 | - | - | - | - |
| PM-4$CeO_2$+Ni[b] | 7 | 98 | 1 | <1 | <1 |
| | 9 | 98 | 1 | <1 | <1 |
| | 10 | 90 | 1 | <1 | <1 |

[a] Reaction conditions: calcined catalyst: 5.4 g; THFA (solvent free): 0.02 mL min$^{-1}$; temperature: 160 °C, $H_2$ pressure: 4.0 MPa, gas-flow rate: 50 mL min$^{-1}$. [b] The catalyst is prepared by physically mixing $CeO_2$ and NiO powders with a molar ratio of 1/80 and subsequent reduction. MTHF = methyltetrahydrofuran.

**Supplementary Table 4**. Selective hydrogenolysis of THFA over various catalysts in a batch reactor.[a]

| Entry[b] | Catalyst | Conversion (%) | Selectivity (%) | | | |
|---|---|---|---|---|---|---|
| | | | 1,5–PDO | 1–butanol | 1–pentanol | MTHF |
| 1 | IM-$CeO_x$/Ni | 19 | 95 | <1 | <1 | <1 |
| 2 | IM-$ReO_x$/Ni | 2 | 63 | 1 | 2 | 22 |
| 3 | IM-$MoO_x$/Ni | 7 | 68 | 2 | 10 | 20 |
| 4 | IM-$VO_x$/Ni | 5 | 62 | 2 | 2 | 8 |
| 5 | IM-$WO_x$/Ni | 2 | 69 | <1 | 11 | 9 |
| 6[c] | 4$CeO_x$/Ni | 35 | 98 | <1 | <1 | <1 |
| 7[c, d] | 4$CeO_x$/Ni | 100 | 98 | <1 | <1 | <1 |
| 8[d] | Ni/$SiO_2$[e] | 6 | <1 | <1 | <1 | 66 |
| 9[d] | Ni/$Al_2O_3$[f] | 15 | <1 | <1 | <1 | 48 |
| 10[d] | Ni/C[g] | 10 | <1 | <1 | <1 | 67 |

[a] Reaction conditions: catalyst, 0.25 g; THFA, 0.5 g; temperature, 160 °C; ethanol, 5 mL; 4.0 MPa $H_2$; 10 h. [b] IM-M`$O_x$/Ni (M`=Re, Mo, V, W, molar ratio of M`/Ni = 1/80) catalysts in entry 1–5 are prepared by impregnation method (IM). [c] 4$CeO_x$/Ni catalyst is prepared by coprecipitation method (CP). [d] 24 h. [e] Ref. 16. [f] Ref. 17. [g] Ref. 18 MTHF = methyltetrahydrofuran

**Supplementary Table 5**. Selective hydrogenation of representative lignocellulose derivates and polyether/ester over $4CeO_x/Ni$ and $96CeO_x/Ni$ catalysts in a batch reactor.

| Substrate | Catalyst | Conversion (%) | Selectivity (%) | | | |
|---|---|---|---|---|---|---|
| 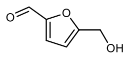 | | | 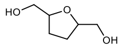 | 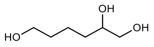 | | |
| (HMF)[a] | $4CeO_x/Ni$ | 100 | 5 | 91 | | |
| | $96CeO_x/Ni$ | 100 | 83 | 8 | | |
| 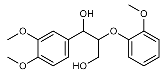 | | | 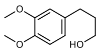 | 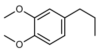 | 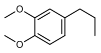 | |
| (VG)[b] | $4CeO_x/Ni$ | 100 | 83 | 4 | 2 | |
| | $96CeO_x/Ni$ | 100 | 26 | 45 | 5 | |
| 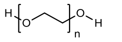 | | | 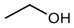 | 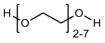 | | |
| (PEG-200)[c] | $4CeO_x/Ni$ | 100 | 85 | 2 | | |
| | $96CeO_x/Ni$ | 100 | 12 | 80 | | |
| 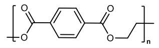 | | | 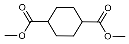 | 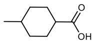 | 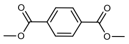 | |
| (PET)[d] | $4CeO_x/Ni$ | 100 | 80 | 12 | 3 | |
| | $96CeO_x/Ni$ | 90 | 9 | trace | 89 | |
| 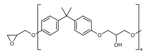 | | | 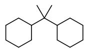 | 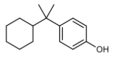 | 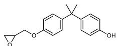 | 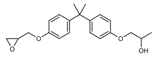 |
| (PBAE)[e] | $4CeO_x/Ni$ | 100 | 33 | 20 | 29 | 2 |
| | $96CeO_x/Ni$ | 70 | trace | trace | 10 | 30 |

[a] catalyst, 0.25 g; HMF, 0.5 g; temperature, 160 °C; ethanol, 5 mL; 4.0 MPa $H_2$; 24 h. [b] catalyst, 0.12 g; HMF, 0.25 g; temperature, 150 °C; ethanol, 5 mL; 6.0 MPa $H_2$; 36 h. [c] catalyst, 0.4 g; PEG-200, 0.5 g; temperature, 160 °C; ethanol, 5 mL; 4.0 MPa $H_2$; 48 h. [d] catalyst, 0.4 g; PET, 0.5 g; temperature, 170 °C; methanol, 10 mL; 4.0 MPa $H_2$; 24 h. [e] catalyst, 0.25 g; PBAE, 0.5 g; temperature, 170 °C; methanol, 10 mL; 4.0 MPa $H_2$; 24 h. HMF = 5–hydroxymethyl furfural. VG = veratrylglycerol–β–guaiacyl ether. PEG = polyethylene glycol. PET = polyethylene terephthalate. PBAE = poly(bisphenol A-co-epichlorohydrin) glycidyl end-capped.

## Supplementary References


1. Wang, G. D., Kong, D. D., Pan, Y. H., Pan, H. B. & Zhu, J. F. Low energy Ar-ion bombardment effects on the $CeO_2$ surface. *Appl. Surf. Sci.* **258**, 2057-2061 (2012).
2. Chen, A., et al. Structure of the catalytically active copper-ceria interfacial perimeter. *Nat. Catal.* **2**, 334-341 (2019).
3. Zheng, X., et al. Highly efficient porous $Fe_xCe_{1-x}O_{2-\delta}$ with three-dimensional hierarchical nanoflower morphology for $H_2S$-selective oxidation. *ACS Catal.* **10**, 3968-3983 (2020).
4. Chia, M., et al. Selective hydrogenolysis of polyols and cyclic ethers over bifunctional surface sites on rhodium-rhenium catalysts. *J. Am. Chem. Soc*. **133**, 12675-12689 (2011).
5. Koso, S., Ueda, N., Shinmi, Y., Okumura, K., Kizuka, T. & Tomishige, K. Promoting effect of Mo on the hydrogenolysis of tetrahydrofurfuryl alcohol to 1,5-pentanediol over $Rh/SiO_2$. *J. Catal.* **267**, 89-92 (2009).
6. Pholjaroen, B., Li, N., Huang, Y., Li, L., Wang, A. & Zhang, T. Selective hydrogenolysis of tetrahydrofurfuryl alcohol to 1,5-pentanediol over vanadium modified $Ir/SiO_2$ catalyst. *Catal. Today* **245**, 93-99 (2015).
7. Chen, K., Mori K., Watanabe, H., Nakagawa, Y. & Tomishige, K. C-O bond hydrogenolysis of cyclic ethers with OH groups over rhenium-modified supported iridium catalysts. *J. Catal.* **294**, 171-183 (2012).
8. Feng, S., Nagao, A., Aihara, T., Miura, H. & Shishido, T. Selective hydrogenolysis of tetrahydrofurfuryl alcohol on $Pt/WO_3/ZrO_2$ catalysts: Effect of $WO_3$ loading amount on activity. *Catal. Today* **303**, 207-212 (2018).
9. Wijaya, H. W., Hara, T., Ichikuni, N. & Shimazu, S. Hydrogenolysis of tetrahydrofurfuryl alcohol to 1,5-pentanediol over a nickel-yttrium oxide catalyst containing ruthenium. *Chem. Lett.* **47**, 103-106 (2018).
10. Lee, J., Xu, Y. & Huber G. W. High-throughput screening of monometallic catalysts for aqueous-phase hydrogenation of biomass-derived oxygenates. *Appl. Catal., B* **140-141**, 98-107 (2013).
11. Soghrati, E., Ong, T. K. C., Poh, C. K., Kawi, S. & Borgna, A. Zeolite–supported nickel phyllosilicate catalyst for C-O hydrogenolysis of cyclic ethers and polyols. *Appl. Catal., B* **235**, 130-142 (2018).
12. Soghrati, E., Kok, Poh, C., Du, Y., Gao, F., Kawi, S. & Borgna A. C-O hydrogenolysis of tetrahydrofurfuryl alcohol to 1,5-pentanediol over bi-functional nickel-tungsten catalysts. *ChemCatChem* **10**, 4652-4664 (2018).
13. Al-Yusufi, M., et al. Efficient base nickel-catalyzed hydrogenolysis of furfural-derived tetrahydrofurfuryl alcohol to 1,5-pentanediol. *ACS Sustainable Chem. Eng.* **10**, 4954-4968 (2022).
14. Wang, Z., et al. Selective hydrogenolysis of tetrahydrofurfuryl alcohol to 1,5-pentanediol over $PrO_x$ promoted Ni catalysts. *Catal. Today* **402**, 79-87 (2022).
15. Yan, H., et al. Construction of stabilized bulk-nano interfaces for highly promoted inverse $CeO_2/Cu$ catalyst. *Nat. Commun.* **10**, 3470 (2019).
16. He, J., Zhao, C. & Lercher J. A. Ni-catalyzed cleavage of aryl ethers in the aqueous phase. *J. Am. Chem. Soc.* **134**, 20768-20775 (2012).
17. Qi, L., et al. Unraveling the dynamic network in the reactions of an alkyl aryl ether catalyzed by $Ni/\gamma$-$Al_2O_3$ in 2-propanol. *J. Am. Chem. Soc.* **141**, 17370-17381 (2019).
18. Song, Q., et al. Lignin depolymerization (LDP) in alcohol over nickel-based catalysts via a fragmentation–hydrogenolysis process. *Energy Environ. Sci.* **6**, 994-1007 (2013).
